# Super-Enhancers and Broad H3K4me3 Domains Form Complex Gene Regulatory Circuits Involving Chromatin Interactions

**DOI:** 10.1038/s41598-017-02257-3

**Published:** 2017-05-19

**Authors:** Fan Cao, Yiwen Fang, Hong Kee Tan, Yufen Goh, Jocelyn Yeen Hui Choy, Bryan Thean Howe Koh, Jiong Hao Tan, Nicolas Bertin, Aroul Ramadass, Ewan Hunter, Jayne Green, Matthew Salter, Alexandre Akoulitchev, Wilson Wang, Wee Joo Chng, Daniel G. Tenen, Melissa J. Fullwood

**Affiliations:** 1 Cancer Science Institute of Singapore, National University of Singapore, Singapore, Singapore; 2 Department of Orthopedic Surgery, National University Health Systems (NUHS), Singapore, Singapore; 3 Oxford BioDynamics Plc, Oxford, England UK; 4 School of Biological Sciences, Nanyang Technological University, Singapore, Singapore; 5 Institute of Molecular and Cell Biology, Agency for Science, Technology and Research (A*STAR), Singapore, Singapore; 6 Yale-NUS Liberal Arts College, Singapore, Singapore; 7 National University Cancer Institute, National University Health System, Singapore, Singapore; 8 Department of Medicine, Yong Loo Lin School of Medicine, National University of Singapore, Singapore, Singapore; 9 Human Longevity Singapore Pte. Ltd., Singapore, Singapore

## Abstract

Stretched histone regions, such as super-enhancers and broad H3K4me3 domains, are associated with maintenance of cell identity and cancer. We connected super-enhancers and broad H3K4me3 domains in the K562 chronic myelogenous leukemia cell line as well as the MCF-7 breast cancer cell line with chromatin interactions. Super-enhancers and broad H3K4me3 domains showed higher association with chromatin interactions than their typical counterparts. Interestingly, we identified a subset of super-enhancers that overlap with broad H3K4me3 domains and show high association with cancer-associated genes including tumor suppressor genes. Besides cell lines, we could observe chromatin interactions by a Chromosome Conformation Capture (3C)-based method, in primary human samples. Several chromatin interactions involving super-enhancers and broad H3K4me3 domains are constitutive and can be found in both cancer and normal samples. Taken together, these results reveal a new layer of complexity in gene regulation by super-enhancers and broad H3K4me3 domains.

## Introduction

Cancer is one of the major causes of mortality and morbidity worldwide. Super-enhancers are large enhancer regions found to drive key oncogenic factors in multiple myeloma, lung cancer, and glioblastoma multiforme^1^. Recent studies have suggested that broad tracts of histone modifications are associated with cancer and cell identity^2–4^. A recent study characterizing the epigenomic properties of super-enhancers in over 80 cell types showed that super-enhancers can be identified through H3K27ac marks, and they are associated with disease-associated genomic variants^2^. In addition, there have been reports that broad H3K4me3 domains are involved in increased transcription elongation and increased enhancer activity at tumor suppressor genes^4^. A major question in understanding super-enhancers and broad H3K4me3 domains is the genes they regulate, and if there are any similarities between the two types of broad domains.

Chromatin interactions are two distal regions of the genome that are brought together in close spatial proximity by protein factors, and can play multiple roles in cancer^5^. Several molecular and genetic approaches have been developed to study three-dimensional (3D) chromosome folding^6, 7^. We exploited published chromatin interactions identified by Chromatin Interaction Analysis with Paired-End Tag Sequencing (ChIA-PET)^8, 9^, which captures genome-wide chromatin interactions bound by a specific protein factor, for data analysis. Two regions of interest were validated using circular chromosome conformation capture (4C), which identifies genome regions that a specific viewpoint interacts with^10, 11^.

The relationship between chromatin interactions and super-enhancers has been studied in the context of cohesin-associated chromatin interactions identified by ChIA-PET^12^. The authors proposed that super-enhancers are wrapped within insulated domains marked by CTCF, together with one or two genes in most cases. However, it is not clear how the presence of super-enhancers involved in chromatin interactions affects their target genes within these insulated domains. Here, we asked how the looping of a super-enhancer to a gene is associated with the gene’s expression level and cell-type specificity.

In this analysis, we studied the transcription regulatory effects and cancer related roles of genes targeted by super-enhancers and broad H3K4me3 domains through proximity or transcription-associated chromatin interactions detected by RNA Polymerase II-associated ChIA-PET data^13^ in K562 chronic myelogenous leukemia cells and MCF-7 breast cancer cells. We associated super-enhancers and broad H3K4me3 domains with chromatin interactions and examined the differences between proximal and distal regulatory elements and their effects on transcription. We found that many proximal super-enhancers not only target oncogenes but also tumor suppressor genes. By comparing super-enhancers with broad H3K4me3 domains, we found that most proximal super-enhancers targeting tumor suppressor genes overlap broad H3K4me3 domains. We confirmed several chromatin interactions in clinical samples and found examples of constitutive transcriptional circuits between cancer and non-cancer samples. Taken together, our findings suggest a role for these broad elements in transcriptional regulation via chromatin interactions.

## Results

### Characterizing proximal and distal super-enhancers

We identified super-enhancers using K562 H3K27ac ChIP-Seq data from ENCODE^14^ following the procedure in Hnisz *et al*.^2^ with some variations (A flowchart of the data analysis procedures is shown for reference in Figure S1). Briefly, peaks were called from the ChIP-Seq data and peaks located within some distance from each other were stitched together. Super-enhancers were identified from these stitched peaks.

**Figure 1:**
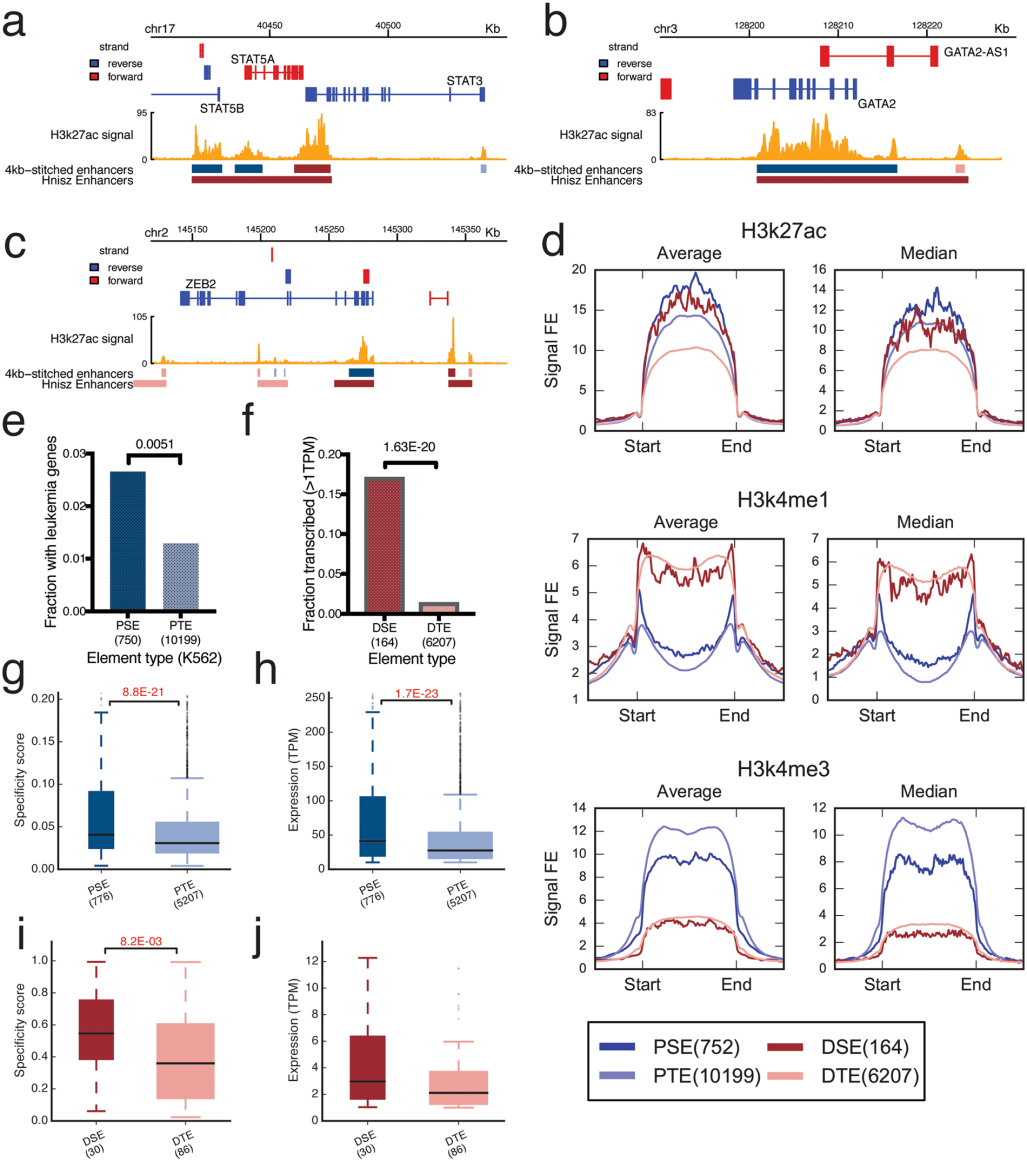
Characterizing proximal and distal super-enhancers. (**a**–**c**) Screenshots of proximal super-enhancers and distal super-enhancers at *GATA2* and *STAT5A*/*B*, and ZEB2 regions. The tracks shown are: (from top to bottom) (1) Genes, (2) K562 H3K27ac ChIP-Seq signal, (3) called typical enhancers and super-enhancers (dark red indicates distal super-enhancers, dark blue indicates proximal super-enhancers, light pink indicates distal typical enhancers and light blue indicates proximal typical enhancers), and (4) Super-enhancers in K562 reported by Hnisz *et al*. (**d**) Histone modifications (H3K27ac, H3K4me1, H3K4me3) at the four types of regulatory elements, including proximal super-enhancers (PSE), proximal typical enhancers (PTE), distal super-enhancers (DSE) and distal typical enhancers (DTE). (**e**) The fraction of proximal elements found at leukemia associated genes. (**f**) Fractions of distal super-enhancers (DSE) and distal typical enhancers (DTE) associated with enhancer transcription. TPM indicates tags per million sequences. (**g**,**h**) The specificities (**g**) and expression levels (**h**) of CAGE clusters at proximal super-enhancers and proximal typical enhancers. All boxplots presented were prepared in the following manner: the black horizontal line indicates the median, the top and bottom of the box indicates the third and first quartile respectively, and the whiskers indicate 1.5* the interquartile range. Widths of boxes are in proportion to the square root of the number of data points in each category and the statistics testing was done using Dunn’s Test. (**i**,**j**) The cell-type specificities (**i**) and expressions (**j**) of enhancer RNAs at distal super-enhancers (DSE) and distal typical enhancers (DTE). See also Figure S2 for MCF-7.

**Figure 2:**
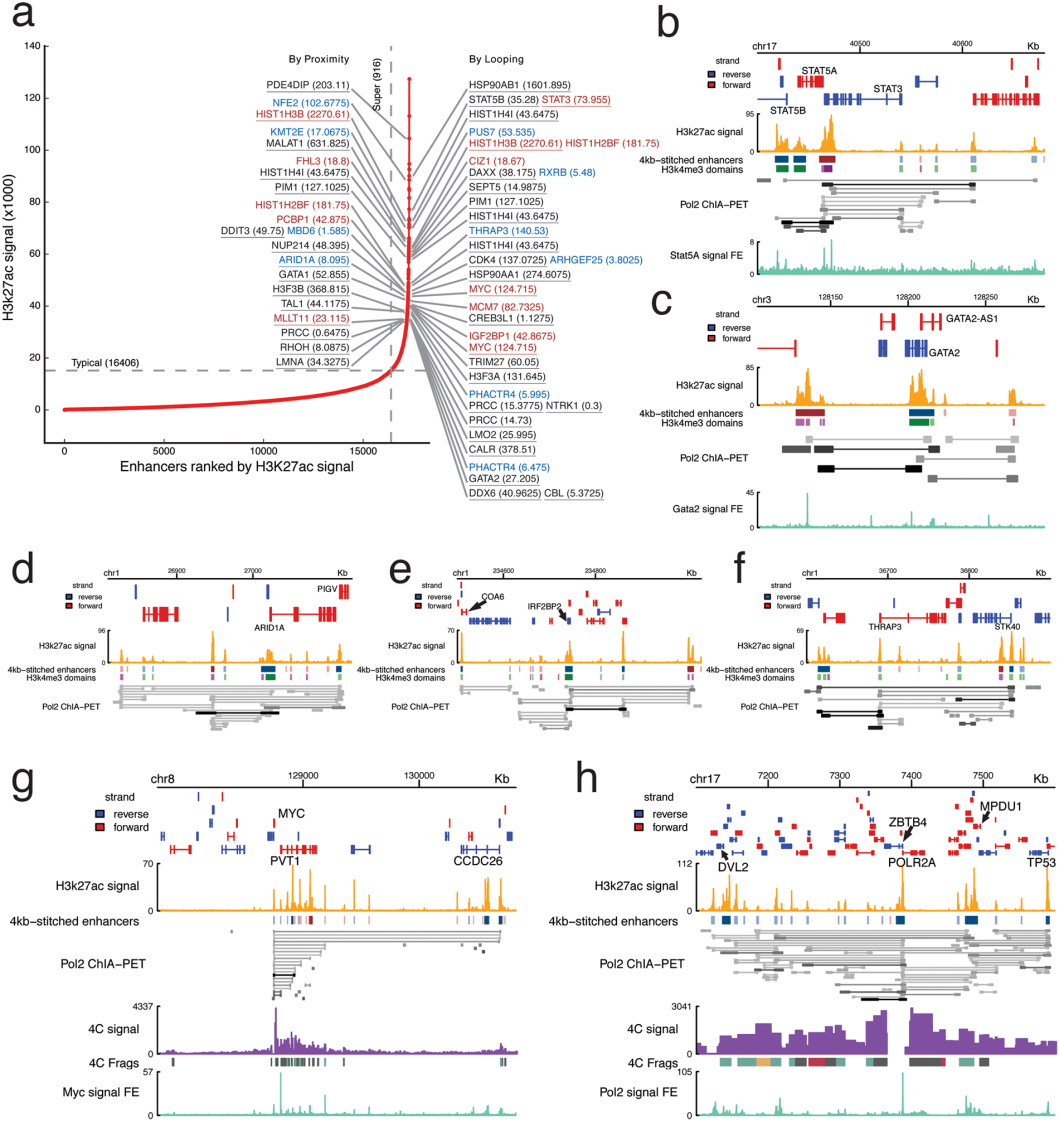
Super-enhancers associated with chromatin interactions. (**a**) Signal rank plot with targeted genes by proximity and by looping. Blue: tumor suppressor genes. Red: oncogenes. Underlined: cancer census genes. (**b**–**f**) Screenshots of super-enhancers and chromatin interactions at *STAT5A*/*B*, *GATA2*, *ARID1A*, *IRF2BP2*, *THRAP3*. (**g**,**h**) Screenshots with 4C-Seq data at *POLR2A* and *MYC*.

As the super-enhancers were very long and close visual inspection of individual super-enhancers suggested that they may be composed of multiple super-enhancers, we used 4 kb windows to merge peaks instead of 12.5 kb used previously (Figs 1a–c and 2a; Table S1). Large proportions of previously identified super-enhancers were located at gene-proximal regions even after removing peaks from promoter regions before stitching^2^. In this study, we retained the peaks found within promoter regions. We classified “enhancers” that are within 4 kb of transcription start sites as “proximal enhancers” and others as “distal enhancers” (Table S1). In total, we identified 752 proximal super-enhancers, 164 distal super-enhancers, 10199 proximal typical enhancers, and 6207 distal typical enhancers.

Several proximal super-enhancers were found to be located at leukemia-associated genes such as *GATA2*, *STAT5A*/*B*, and *ZEB2* (Fig. 1a–c; Table S1), as expected of super-enhancers found in a leukemia cell line. From COSMIC cancer Gene Census^15^, which is a manually curated database of cancer associated genes, we extracted potential leukemia associated genes. We found 18 proximal super-enhancers and 75 proximal typical enhancers located near leukemia associated genes. Proximal super-enhancers were significantly enriched at leukemia associated genes compared to proximal typical enhancers (Fisher’s Exact Test, p = 0.0051) (Fig. 1e).

Proximal and distal enhancers showed different histone marks. Proximal enhancers showed higher H3K4me3 signals, whereas distal enhancers showed higher H3K4me1 signals (Fig. 1d). This is in line with the notion that H3K4me3 is associated with promoters and H3K4me1 is associated with enhancers^16^. We examined the capped analysis of gene expression data (CAGE) from the FANTOM 5 project^17, 18^ to obtain the expression levels of genes across a wide range of cell types. We found that transcription of genes at proximal super-enhancers was more cell-type specific (Fig. 1g) and had higher expression levels (Fig. 1h) than transcription of genes at proximal typical enhancers. A larger proportion of distal super-enhancers were found to be transcribed than distal typical enhancers as measured by the bi-directionally transcribed CAGE clusters^18^ (Fig. 1f). The enhancer RNAs transcribed at distal super-enhancers were more cell-specific (Fig. 1i) and have slightly higher transcription levels than those transcribed at distal typical enhancers (Fig. 1j). Similar results were found in MCF-7 cell line (Figure S2).

### Super-enhancers connect with target genes via long-range chromatin interactions

To find out how regulatory elements link with each other, we overlapped the regulatory elements with chromatin interactions bound by RNA polymerase II (Pol II) from Pol II ChIA-PET data^13^ (Fig. 2a).

We annotated the genes using two different databases: (1) the lists of oncogenes and tumor suppressor genes from Tumor Suppressor and Oncogene (TUSON) Explorer^19^, a computational method that analyzed mutational signatures in tumor-normal pairs to predict how likely a gene is a tumor suppressor gene or an oncogene; (2) COSMIC Cancer Gene Census^15^.

Many super-enhancers were associated with cancer-associated genes, including tumor suppressor genes and oncogenes, through both proximity and looping (Fig. 2a). Specific examples of super-enhancer looping to target genes are shown in Fig. 2b–f. Similar results can be seen in MCF-7 upon analysis with RNA polymerase II ChIA-PET data in MCF-7 (Figure S2).

Using 4C at *POLR2A* promoter and Pol II ChIP-Seq^14^, we validated several chromatin interactions from *POLR2A* promoter to other proximal super-enhancers and proximal typical enhancers which were also enriched in RNA polymerase II (Fig. 2g). Super-enhancer regulating leukemia associated genes such as *STAT5A* (Fig. 2b) and *GATA2* (Fig. 2c) also showed enrichment of *STAT5A* and *GATA2*, respectively. Using 4C at *MYC* promoter and ChIP-Seq of c-MYC^14^, we showed that the *MYC* promoter was regulated by super-enhancers and typical enhancers that were occupied by c-MYC (Fig. 2h).

### Super-enhancers are more involved in chromatin interactions than typical enhancers

**Figure 3:**
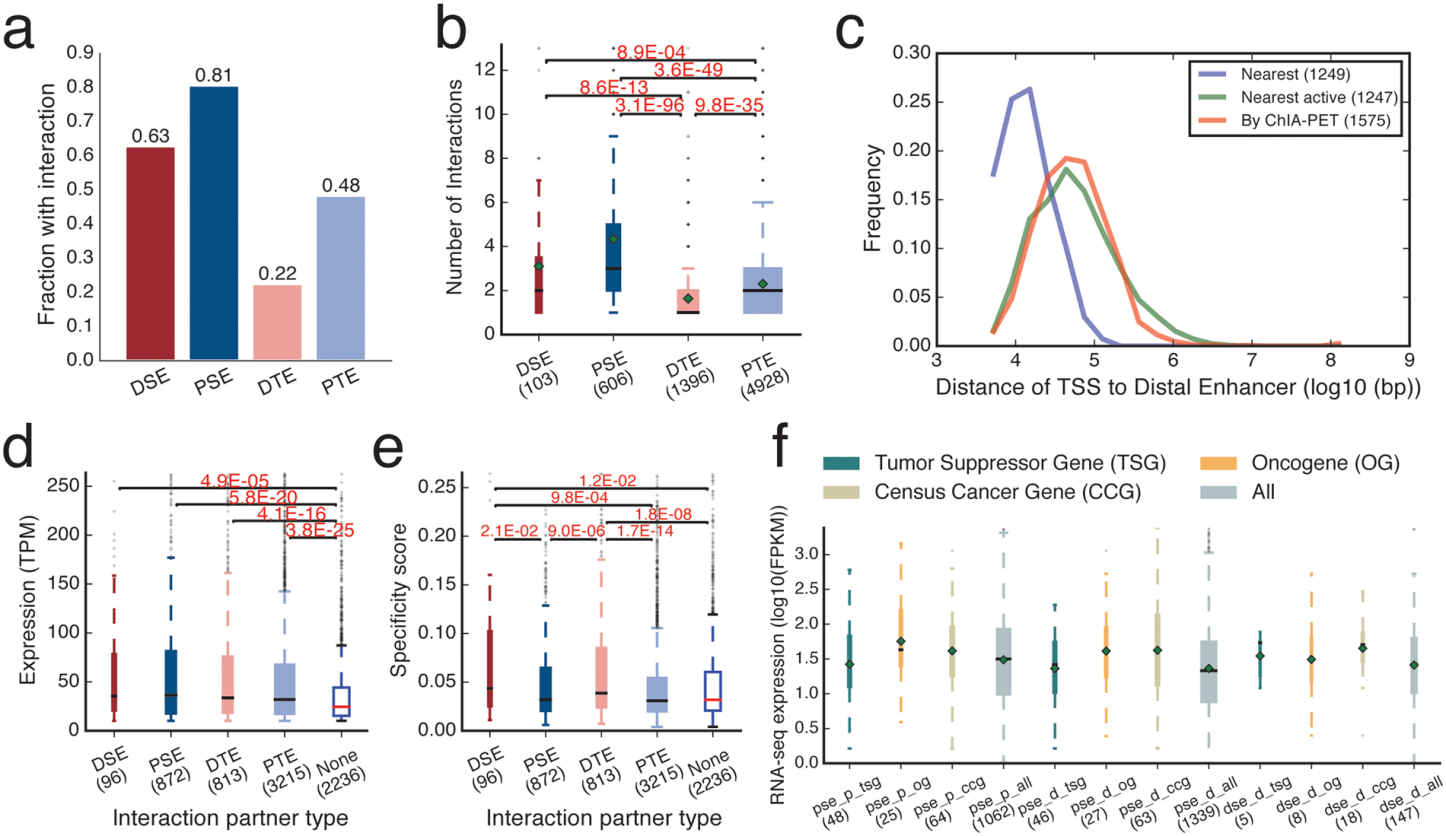
(**a**) Fractions of the four types of elements associated with at least one chromatin interaction. (**b**) Boxplot for the number of interactions each type of element has (only interacting elements were included). (**c**) Distribution of distances of the nearest TSS (blue), nearest active TSS (green), and TSSes through chromatin interactions (red) to the center of distal super-enhancers. (**d**,**e**) The expression levels (**d**) and cell-type specificity scores (**e**) of CAGE clusters at proximal elements that are connected to the four types of elements as indicated on the x-axis. “None” indicates the set of CAGE clusters located at non-interacting proximal elements. (**f**) The expression levels of tumor suppressor genes (tsg), oncogenes (og), and cencus cancer genes (ccg) targeted by broad domains through proximity (pse_p) and looping (pse_d and dse_d) measured by RNA-Seq. Data shown is for K562. See Figure S3 for MCF-7.

By looking at the Pol II chromatin interactions between these four types of enhancers, we found that 63% of distal super-enhancers, 81% of proximal Super-enhancers, 22% of distal typical enhancers, and 48% of proximal typical enhancers were involved in such chromatin interactions (Fig. 3a; Table S1). For those involved in chromatin interactions, it appeared that super-enhancers were generally involved in more chromatin interactions than typical enhancers (Fig. 3b).

As super-enhancers usually span larger regions than typical enhancers, the differences in frequency of involvement in chromatin interaction might be due to the differences in enhancer sizes. For each of the four categories, we calculated the average enhancer size of the category. The maximum average size was then obtained. For enhancers in each category, they were extended by the difference between the average size of its category and the maximum average size of the four categories. The extension did not affect the interaction frequencies or the number of associated chromatin interactions significantly (Figure S5a,b). However, when each enhancer’s number of chromatin interactions was normalized against its total Pol II signal measured by the ChIA-PET dataset, interacting typical enhancers showed more chromatin interactions per Pol II signal than interacting super-enhancers (Figure S5c).

These observations led us to test whether super-enhancers would also show higher association with chromatin interactions detected by a method that is not reliant on ChIP signal. We therefore performed the association analyses with high-resolution Hi-C data^20^. The result still showed that a larger proportion of super-enhancers were associated with chromatin interactions compared with typical enhancers (Figure S5d,e). The differences were still large even after extending the enhancers to eliminate the size differences between different categories (Figure S5f,g). These results indicated that super-enhancers were inded more associated with chromatin interactions. It should be noted, however, that differences in the number of associated chromatin interactions were not observed when using Hi-C data.

Next, we associated interacting super-enhancers and typical enhancers to genes through chromatin interactions (Table S1). Some super-enhancers have multiple target genes via chromatin interactions, and some can “skip” genes, indicating that the target genes could not be simply predicted by using the heuristic of the “nearest gene” (Fig. 3c; Table S1). This is consistent with previous observations that enhancers can loop to distant target genes^11, 18^. We found that target genes could be predicted by the heuristic of the “nearest active gene”, consistent with previous findings^21^, but this is limited to only one or two genes and might not give the full extent of genes that an enhancer can loop to.

We examined the effect of chromatin interactions on the expression of genes using CAGE clusters at proximal enhancers. Genes near proximal enhancers that were involved in chromatin interactions showed significantly higher expression levels than those not involved in chromatin interactions, regardless of whether the chromatin interaction was to super-enhancers or to typical enhancers (Fig. 3d; Figure S6a). Genes at proximal enhancers that were connected to distal enhancers displayed higher cell-type specificities than genes at proximal enhancers that were connected to other proximal enhancers or not involved in chromatin interactions (Fig. 3e; Figure S6b).

Interestingly, in addition to observing super-enhancers targeting oncogenes, such as *c-MYC*, we also observed super-enhancers that were either near tumor suppressor genes or connected to tumor suppressor genes through chromatin interactions (Figs 2a and 3f; Tables S1 and S2). Several chromatin interactions could even connect tumor suppressor genes and oncogenes (Table S1). These results are consistent with previous findings showing that super-enhancers could also regulate tumor suppressor genes^22^.

We examined the expression levels of tumor suppressor genes and oncogenes that were regulated by super-enhancers. Tumor suppressor genes showed lower expression levels than oncogenes (Fig. 3f). The observation suggested that although some tumor suppressor genes were regulated by super-enhancers, tumor suppressor genes in general were not as highly activated as oncogenes in cancer. Similar results were found in MCF-7 breast cancer cells (Figure S3).

### Broad H3K4me3 domains connect with target genes via chromatin interactions

**Figure 4:**
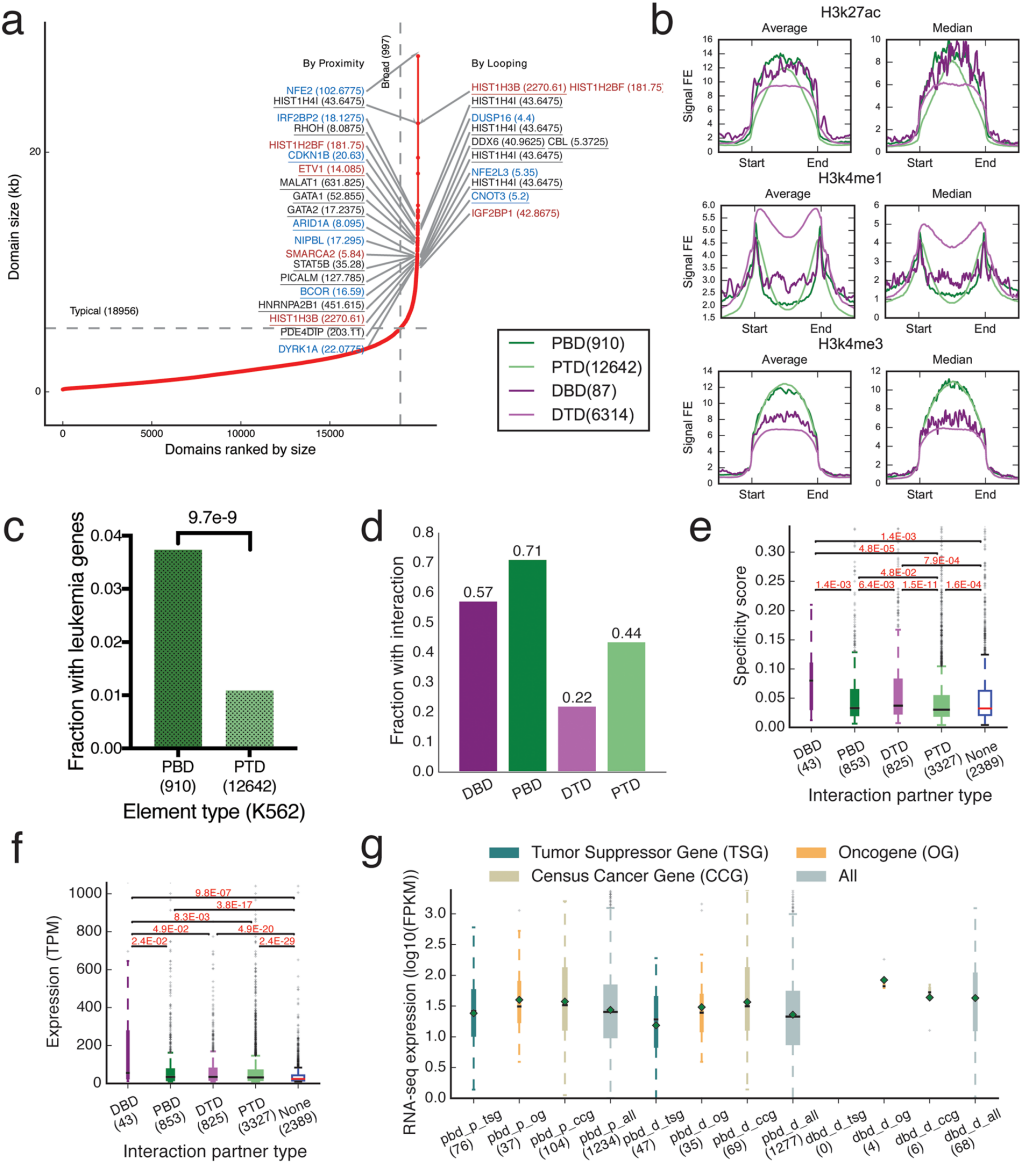
Analysis of broad H3K4me3 peaks and chromatin interactions. (**a**) Rank of H3K4me3 peaks by size and the cancer associated genes targeted by some broad domains. (**b**) Some histone modifications at the four types of H3K4me3 domains. (**c**) The fraction of proximal broad and typical domains located near leukemia associated genes. p-value is produced Fisher’s Exact Test. (**d**) The fraction of each type of elements by H3K4me3 involved in chromatin interactions. (**e**,**f**) The cell-type specificity scores (**e**) and expression levels (**f**) of CAGE clusters at proximal H3K4me3 elements that are connected to the four types of H3K4me3 elements as indicated on the x-axis. “None” indicates the set of CAGE clusters located at non-interacting proximal elements. (**g**) The expression levels of tumor suppressor genes (tsg), oncogenes (og), and cencus cancer genes (ccg) targeted by broad domains through proximity (pbd_p) and looping (pbd_d and dbd_d) measured by RNA-Seq. Data shown is for K562. See Figure S4 for MCF-7.

Broad H3K4me3 peaks were called using MACS2^23^ with the option “–broad”^14^. A total of 19953 peaks were obtained. We ranked the H3K4me3 peaks by their lengths and took the top 5% as broad H3K4me3 domains and the rest as typical domains^3^ (Fig. 4a). Using the same procedure as for super-enhancers, we classified the H3K4me3 domains into proximal and distal domains (Table S3). 91.3% broad H3K4me3 domains and 66.7% of typical H3K4me3 domains are proximal to genes (Fig. 4b). In K562 cells proximal broad H3K4me3 domains were enriched at leukemia associated genes, which is similar to our observations with super-enhancers (Fig. 4c). Larger proportions of broad H3K4me3 domains were involved in chromatin interactions compared to typical H3K4me3 domains (Fig. 4d). Genes at proximal H3K4me3 domains showed higher expression levels when involved in chromatin interactions (Fig. 4f). Higher cell specificities were observed for genes targeted by distal H3K4me3 domains through chromatin interactions (Fig. 4e). MCF-7 data showed similar patterns (Figure S4).

### A subset of super-enhancers and broad H3K4me3 domains overlap

Using oncogenes and tumor suppressor genes from TUSON explorer^19^, Chen *et al*. showed that despite the large overlap between genes assigned to broad H3K4me3 domains and genes assigned to super-enhancers, only genes exclusively assigned to broad H3K4me3 domains were enriched of tumor suppressor genes^4^. In this study, we wished to investigate the differences in regulation among regions with both super-enhancers and broad H3K4me3 domains, regions with only super-enhancers, and regions with only broad H3K4me3 domains.

**Figure 5:**
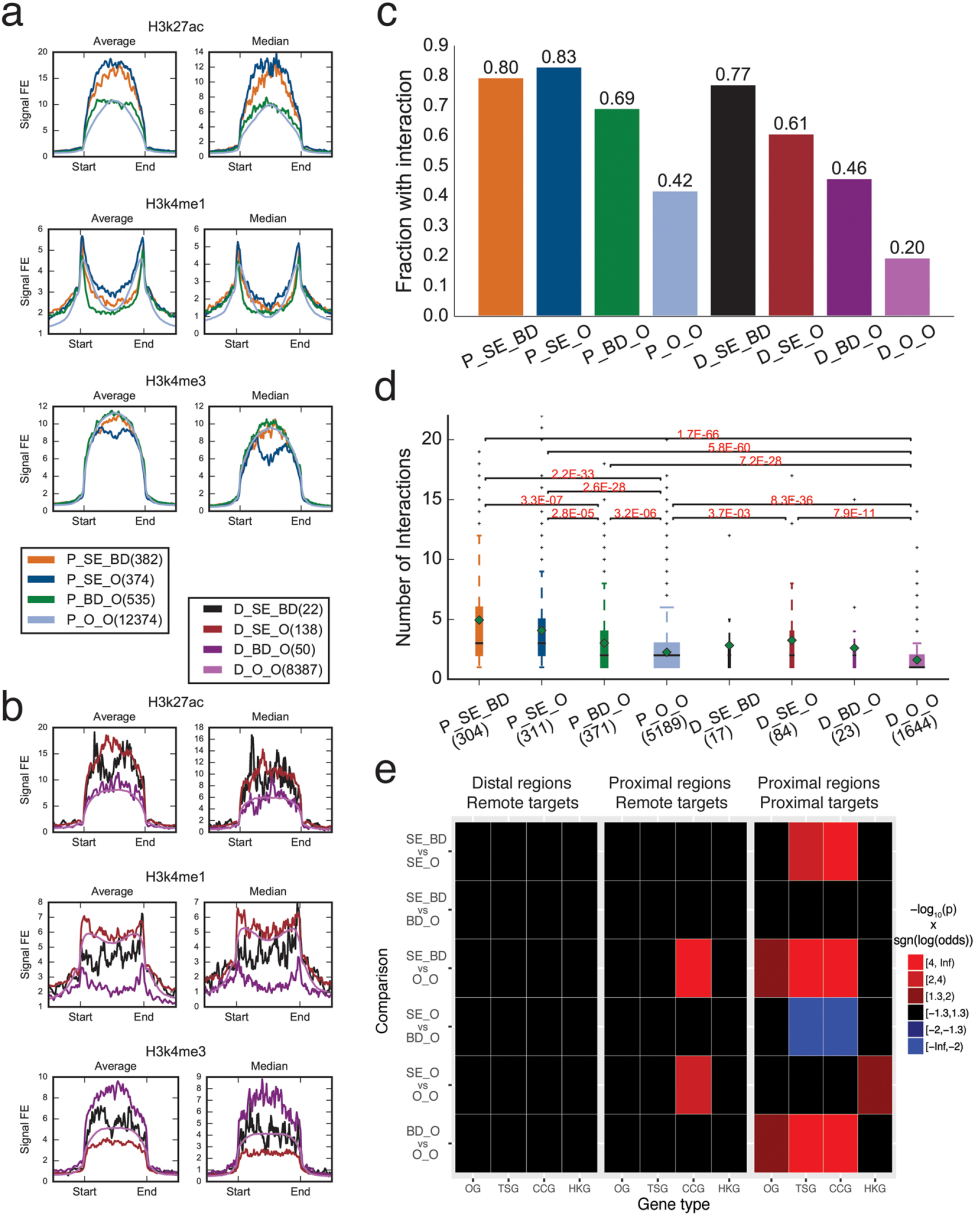
Overlap between super-enhancers and broad H3K4me3 domains in K562 cells. (**a**) Histone modifications at proximal regions with both super-enhancers and broad H3K4me3 domains (P_SE_BD), only super-enhancers (P_SE_O), only broad H3K4me3 domains (P_BD_O), and neither (P_O_O). (**b)** Histone modifications at distal regions with both super-enhancers and broad H3K4me3 domains (D_SE_BD), super-enhancers only (D_SE_O), broad H3K4me3 domains only (D_BD_O), and neither (D_O_O). (**c**) The fraction of different regions involved in chromatin interactions. (**d**) Boxplot of number of chromatin interactions each type of regions is involved in. (**e**) Pairwise comparison of enrichment at oncogenes (OG), tumor suppressor genes (TSG), COSMIC census cancer genes (CCG), and housekeeping genes (HKG) between different distal regions through looping (left), and different proximal regions through looping (middle) and proximity (right). Comparison was performed using Fisher’s Exact Test followed by Holm-Šídák correction for multiple testing. The color indicates the product of negative log-transformed p-values and sign of the log-transformed corresponding odds ratio.

Many super-enhancers showed overlap with broad H3K4me3 domains (Figures S10a ,b and S12a,b). We obtained the regions with both super-enhancers and broad H3K4me3 domains (SE_BD), regions with super-enhancers but not broad H3K4me3 domains (SE_O), regions with broad H3K4me3 domains but not super-enhancers (BD_O), and regions with typical enhancers or typical H3K4me3 domains (O_O). We then divided them into proximal (with P_ prefix) and distal (with D_ prefix) as mentioned above. The SE_BD regions showed combined enrichment of both H3K27ac and H3K4me3 histone marks, whereas the SE_O regions were relatively low in H3K4me3 occupancy and BD_O regions were relatively low in H3K27ac occupancy. BD_O regions also showed the lowest occupancy of H3K4me1 histone mark (Fig. 5a,b; Figure S7a,b).

We looked at whether these different types of regions had different effects on expressions of target genes using CAGE data^17^. CAGE clusters at proximal SE_BD, SE_O and BD_O regions showed higher expression levels and higher specificities compared to CAGE clusters at regions with neither super-enhancer nor broad H3K4me3 domains in both K562 (Figure S10c,d) and MCF-7 (Figure S12c,d) cells, while the differences among proximal SE_BD, SE_O and BD_O regions were not consistently detected in K562 and MCF-7 cells.

Different types of regions showed different levels of association with chromatin interactions. In general, SE_BD and SE_O regions showed highest level of association with chromatin interactions where SE_O regions is slightly lower, followed by BD_O regions and O_O regions in decreasing order (Fig. 5c; Figure S7c). The same order was observed in terms of number of chromatin interactions each interacting region was involved in (Fig. 5d; Figure S7d). This suggested that regions with both high H3K27ac occupancy and broad H3K4me3 coverage represented a set of strong regulatory regions that were able to engage in high degrees of looping.

We did not observe any consistent differences in the expression levels and specificities of CAGE clusters targeted by the different types of regions through looping in both K562 and MCF-7 data (Figures S10e –h and S12e–h).

We then investigated whether any type of region was more likely to target oncogenes and/or tumor suppressor genes from TUSON explorer^19^ and/or COSMIC census cancer genes^15^ through either proximity or looping (Fig. 5e; Figure S7e). Housekeeping genes^24^ were also included in the analysis as controls. Through looping, distal regulatory regions with super-enhancers or broad H3K4me3 regions or both did not show enrichment in targeting the four groups of genes compared to other distal regions. Proximal SE_BD and proximal SE_O regions showed enrichment in targeting COSMIC census cancer genes through looping in K562 cells (Fig. 5e) but not in MCF-7 cells (Figure S7e). We then investigated whether any type of regulatory element is more likely to be found near the genes in the four gene sets. Interestingly, proximal SE_O regions did not show any enrichment, except for housekeeping genes in K562 cells, compared with proximal O_O regions. In contrast, proximal BD_O regions showed significant higher association with tumor suppressor genes and COSMIC census cancer genes compared to proximal SE_O and O_O regions in both K562 and MCF-7 cells. Compared to proximal SE_O and O_O regions, proximal SE_BD regions showed higher association with COSMIC census cancer genes in both K562 and MCF-7 cells and tumor suppressor genes in K562 cells. These observations suggested that in general, proximal regulatory regions with broad H3K4me3 domains, are more likely to be located at cancer related genes, regardless of whether super-enhancers are present.

The expression levels of oncogenes, tumor suppressor genes, and COSMIC census cancer genes targeted by the different types of regulatory regions through looping or proximity did not show apparent patterns. As previously observed, tumor suppressor genes generally showed lower expression levels than oncogenes. Consistent with the enrichment analysis, most of the tumor suppressor genes (38 out of 49 in K562 cells) and COSMIC census cancer genes (51 out of 64 in K562 cells) located at proximal super-enhancer are located at regions with both super-enhancers and broad H3K4me3 domains (Figures S11c and S13c), whereas those targeted through looping did not show almost equal distribution (Figures S11a ,b and S13a,b).

Taken together, these results suggest that the enrichment of proximal super-enhancers at tumor suppressor genes and COSMIC census cancer genes through proximity is mainly due to the co-localization with broad H3K4me3 domains, suggesting a leading role of broad H3K4me3 domains in regulating cancer associated genes in K562 and MCF-7 cells.

### Chromatin interactions can be found in clinical samples

We examined whether super-enhancer and broad H3K4me3 domain-associated chromatin interactions can be found in clinical samples. We used Oxford Biodynamics’ EpiSwitch™ platform to investigate two interaction regions, one at oncogene *MYC*, and the other at tumor suppressor *TP53* in K562 cells and in clinical samples. The EpiSwitch method follows similar steps as in conventional 3C, with several modifications such as including genetically modified TaqI for chromatin digestion and incubation with high efficiency DNA ligase to increase the efficiency of the method, as described previously^25, 26^.

**Figure 6:**
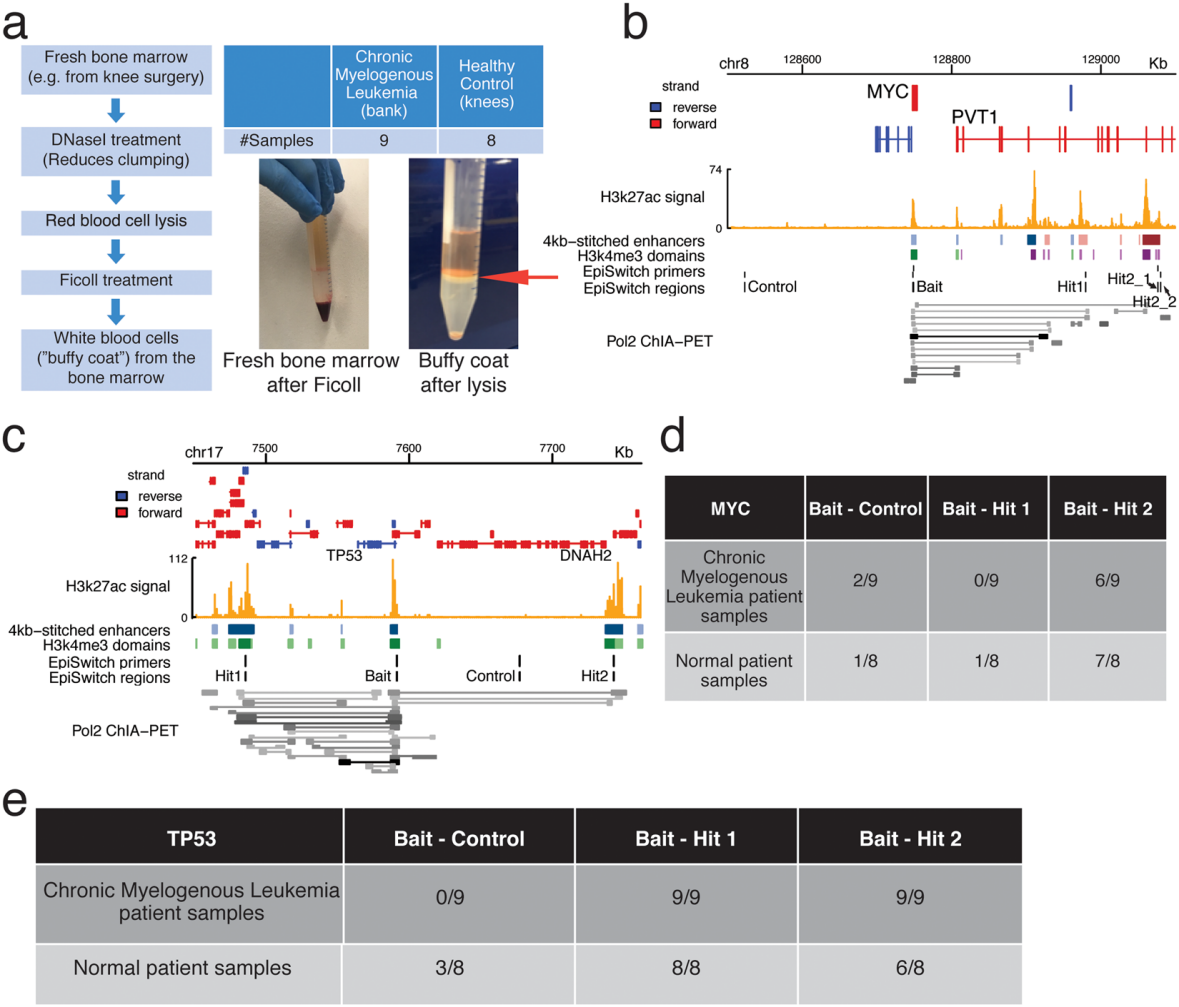
Constitutive circuits in cancer involving super-enhancers and broad H3K4me3 domains. (**a**) Flowchart of bone marrow preparation for EpiSwitch™ analyses, with images showing fresh bone marrow after Ficoll and buffy coat after lysis (the buffy coat is marked by a blue arrow). (**b**) Genome Browser screenshot of the *MYC* locus, showing EpiSwitch™ bait and hit regions. (**c**) Genome Browser screenshot of the *TP53* locus, showing EpiSwitch™ bait and hit regions. (**d**,**e**) Chromatin interactions at the *MYC* locus (**d**) and *TP53* locus (**e**) were screened with EpiSwitch™, together with negative control loci (no chromatin interaction), using CML and non-CML bone marrow samples. “2/9” indicates that of the 9 samples examined, 2 had detected interactions.

After validating the EpiSwitch method in K562 cells (Supplementary Text), we performed this method with peripheral blood samples and bone marrow samples. In the case of bone marrow samples, we obtained white blood cells from the buffy coat (Fig. 6a). The details of the samples used are provided in Table S5.

In K562 cells, one interaction connected *MYC* to a typical enhancer (bait-hit1), and another interaction connected *MYC* to a distal super-enhancer that overlapped with a broad H3K4me3 domain (bait-hit2) (Fig. 6b, Figure S8a). Within the *TP53* locus, two interactions connected the *TP53* promoter, which harbors both a proximal super-enhancer and a proximal broad H3K4me3 domain, to two other regions that were also occupied by both proximal super-enhancers and broad H3K4me3 domains (bait-hit1, bait-hit2) (Fig. 6c, Figure S8a). These chromatin interactions were also present in ChIA-PET data^13^.

We examined whether we could detect the chromatin interactions in patient samples. Some chromatin interactions at *MYC* and *TP53* could be detected in bone marrow and peripheral blood samples (Fig. 6d,e and Figure S8b,c). Not all chromatin interactions that we observed in K562 cells were detected in clinical samples such as the case of bait-hit 1 at the *MYC* locus (Fig. 6d, Figure S8b), indicating that there are differences between cell lines and patient samples and future research to dissect chromatin interactions in cancer will need to include experiments with patient samples.

Moreover, we found that the chromatin interactions tested in the patient samples were similar between cancer and non-cancer samples (Fig. 6d,e, Figure S8b,c). In addition, we note that we detected examples of individual heterogeneity in chromatin interactions (Fig. 6d,e and Figure S8b,c), alongside detection of individual heterogeneity in enhancers as marked by H3K27ac marks (Figure S9c), DNA (Table S6) and RNA (Figure S9b). While we could detect individual heterogeneity in these different cellular processes, we could not detect any correlation between them (Table S7). However, we cannot rule out the possibility of negative results arising from detection limitations or from limited sample sizes (Supplementary Text, Figure S8).

## Discussion

Here, we report a distinction between proximal and distal stretched regulatory genomic elements, and demonstrated an association between them and chromatin interactions. The observation of chromatin interactions connecting these domains with target genes increases the complexity of gene regulatory circuits.

While previous investigations reported super-enhancer-associated control of oncogenes and broad H3K4me3 peaks at tumor suppressor genes, here we show overlap in these two types of elements, suggesting that certain genomic elements could have both roles. This result also explains why we observed that super-enhancers can loop to or locate at not only oncogenes but also tumor suppressor genes, among other genes. While a previous report gave one example of a tumor suppressor gene regulated by a super-enhancer^22^, here we showed through a genome-wide search that the phenomenon of tumor suppressor gene association with super-enhancers is common. These tumor suppressor genes associated with proximal super-enhancers are generally also associated with proximal broad H3K4me3 domains, suggesting an overlap in function of proximal super-enhancers and proximal broad H3K4me3 domains. Subsequent analyses suggested a dominant role of proximal broad H3K4me3 domains in regulating tumor suppressor genes and, more generally, cancer associated genes over proximal super-enhancers in the two cancer cell lines studied.

In addition, genes involved in chromatin interactions showed higher expression levels, regardless of the identity of their looping partners. Genes interacting with distal enhancers showed higher cell type specificities compared with genes interacting with other types of regulatory elements. We observed that protein products of some cancer associated genes targeted by super-enhancers showed enrichment at the super-enhancers, which might form auto-regulatory loops. Further work would be needed to confirm and identify such auto-regulatory loops.

Super-enhancer-associated and broad H3K4me3 domain-associated chromatin interactions can be detected in human clinical samples. While many previous chromatin interaction analyses have employed cell line models, several groups are starting to explore chromatin interactions in patient cancers^27, 28^. We validated examples of enhancer looping to *MYC* and *TP53* by EpiSwitch™ and showed these looping patterns are similar between leukemia and non-leukemia samples. We speculate that constitutive chromatin interactions involving super-enhancers and broad H3K4me3 domains may be “hijacked” by cancer cells to drive stable, high gene expression of mutated genes in cancer. Further study would be needed to confirm the speculation and elucidate the functional mechanisms if the speculation is confirmed. Our experiments interrogate chromatin interactions in the context of a set of patient samples, and set the stage for future studies on patient samples. We note there exists individual heterogeneity even between the same type of sample (which may also be the result of detection limits of methods used), making it necessary to investigate large numbers of samples to dissect out the possible roles of chromatin interactions in health and disease.

Chromatin interaction aberrations arising from genetic abnormalities have been observed previously in leukemia^27^. Detailed comparison of the chronic myelogenous leukemia patient samples with non-leukemic controls will be needed to characterize novel cancer-specific super-enhancer interaction circuits. Alterations in chromatin interactions might underlie the transition from chronic phase to accelerated phase and eventually to blast crisis in chronic myelogenous leukemia and would be valuable to study further.

Comparison of the peripheral blood and bone marrow samples may reveal cell-type specific chromatin interactions. In particular, bone marrow cells tend to be less mature, while peripheral blood tends to comprise large population of granulocytes. Chromatin interaction analysis of additional patient samples, together with fractionation of blood samples, will be needed to explore these possibilities.

In conclusion, this research sets the stage for further elucidation of super-enhancers and broad H3K4me3 domains involved in chromatin interactions, and investigation into the chromatin interaction profile of more patient samples, by revealing a new level of complexity in gene regulation.

## Methods

### Super-enhancer characterization

The list of ChIP-Seq libraries used in this study are detailed in Supplementary Table 4.

K562 and MCF-7 H3K27ac ChIP-Seq and matched input raw sequencing data were obtained from ENCODE^14^. The sequences were mapped to hg19 assembly using Bowtie2 with the option –n 1, indicating that we allowed 1 mismatch in the seed alignment. PCR duplicates were removed using ‘samtools rmdup‘. Reads that fall within the ENCODE consensus blacklisted regions were removed. The two replicates were then combined and peaks were called from the combined dataset using MACS2 (version 2.1.0.20150731) with option “-p 1e-8”. A custom script was used to stitch the peaks using 4 kb as a window, and compute the treatment and control signal for each stitched peak (all scripts are in this link: https://bitbucket.org/cao_fan/sein). The formula used to normalize and compute signal mimics that of ROSE package. The purpose of the custom script was to speed up the computation process. After the treatment and control signals calculated for each stitched peak, the R script from ROSE package was used to identify the set of super-enhancers. In summary, super-enhancers were identified as the highest regions of H3K27ac occupancy from ChIP-Seq data.

Next, we assigned super-enhancers to genes. Gencode V19 annotation was used to obtain the set of transcription starting sites. Transcripts of biotypes Mt_rRNA, Mt_tRNA, Mt_tRNA_pseudogene, miRNA, miRNA_pseudogene, misc_RNA, misc_RNA_pseudogene, rRNA, rRNA-pseudogene, scRNA_pseudogene, snRNA, snRNA_pseudogene, snoRNA, snoRNA_pseudogene, tRNA, tRNA_pseudogene, tRNAscan, retained_intro, TEC, and disrupted_domain were removed. The stitched peaks that are within 4 kb of any transcription start site were annotated as proximal; the rest were termed distal.

Oncogenes and tumor suppressor genes were annotated following Davoli *et al*., using the genes with Tumor Suppressor and Oncogene Explorer (TUSON) p-value smaller than 0.01^19^.

### Chromatin interaction calling from ChIA-PET data

Two sets of previous called chromatin interactions from saturated K562 and MCF-7 ChIA-PET datasets were obtained from GEO (GSM832465). Only chromatin interactions that were found in both replicates were used for the study. The overlap was found using the “pairToPair” command from BEDTools.

### Expression analyses

Annotated CAGE clusters with normalized expression values were downloaded from the FANTOM 5 project website (http://fantom.gsc.riken.jp/5/). The grouping facets were obtained from Andersson *et al*.^18^. The expression of a cluster in a facet was obtained by taking the mean of the cluster’s expressions in all the samples assigned to that facet. We obtained 367 samples (including 4 K562 samples), and grouped them into 70 facets with K562 as an independent facet. In other words, we obtained the expression levels of genes from different cell types from the CAGE data, including K562 expression levels. This was used to calculate the expression level and cell-specificity of the genes associated with super-enhancers and typical enhancers.

The specificity of a cluster was calculated using

$${\rm{Specificity}}({\rm{X}})=1-({\rm{entropy}}({\rm{X}})/\,\mathrm{log}\,2({\rm{N}})),$$

where X is the vector of expression values of the cluster in across all facets, and N = |X|. The definition of specificity is identical to that used by Andersson *et al*.^18^.

RNA-Seq quantification for K562 was downloaded from ENCODE (ENCSR109IQO, ENCSR000AEN)^14^.

### Broad H3K4me3 peak analysis

Broad peaks were called from K562 and MCF-7 H3K4me3 ChIP-Seq data downloaded from ENCODE^14^ using MACS2^23^ (version 2.1.0.20150731) with –broad option. The “gappedPeak” file was used in the subsequent analysis. The flanking “broad” regions were trimmed off. The resulting gapped peaks were then ranked by size and top 5% were taken as broad H3K4me3 domains, others as typical H3K4me3 domains. These domains were then separated into proximal and distal based on whether they are within 4 kb of any transcription starting sites.

### Enrichment analysis

The H3K27ac enhancers and H3K4me3 domains produced were merged. The resulting regions with broad H3K4me3 domains and super-enhancers are annotated as SE_BD regions, those with only super-enhancers were annotated as SE_O regions, those with only broad H3K4me3 domains were annotated as BD_O regions, and the others were annotated as O_O regions. For SE_BD, SE_O and BD_O regions, those contain at least one proximal broad H3K4me3 domain or one proximal super-enhancer are considered as proximal and others are distal. For O_O regions, those contain at least one proximal typical enhancer or one proximal typical H3K4me3 domain were considered as proximal and the others were distal.

For the enrichment analysis on remote targets through chromatin interactions, the total number of each type of regions considered were those involved in chromatin interactions. For the enrichment analysis on proximal targets, the total number of each type of regions were the actual number of the regions. As the list of housekeeping genes contains 3804 genes, we repeatedly sampled 500 genes from the list and make the counts for 1000 times. The average counts were used as the numbers for housekeeping genes. The enrichment analyses were performed using Fisher’s Exact Test followed by Holm-Šídák correction.

### Cell culture

K562 cells were a kind gift from Daniel Tenen’s lab in the Cancer Science Institute. They were cultured in RPMI-1640 supplemented with 10% FBS at 37 °C, 5% CO_2_ with 1x penicillin-streptamycin.

### Circular Chromosome Conformation Capture (4C)

4C-seq was performed as previously described with some modifications^29^. In brief, 4 × 10^7^ cells were harvested and crosslinked with 1% formaldehyde for 10 min at room temperature with rotation. The crosslinking was quenched by glycine for 5 min at room temperature with rotation. Following SDS and Triton X-100 permeabilization, nuclei were digested with HindIII-HF (NEB) overnight. Following proximity ligation, reverse cross-linking and DNA purification, the circular DNA was digested with DpnII (NEB) 37 °C overnight, and circularized. The 4C-seq library was generated by performing nested inverse PCR using Phusion DNA polymerase (Thermo Scientific) with the primers listed in Table S8a. The cycling condition of the 1^st^ PCR was: 98 g condition of the 1inked with 1% formaldehyde for 10 min at room temperature with rotation. The crosslinking was quenchension at 72 °C for 60 s. 10% of the 1^st^ PCR product was used for the 2^nd^ PCR. The cycling condition for the 2^nd^ PCR was: 98cling condition for the 2 with 1% formaldehyde for 10 min at room temperature with rotation. The crosslinking was quenchension at 72 °C 72 °C for 60 s. The 4C-seq library was purified by 4–20% gradient TBE PAGE gel (ThermoFisher Scientific) and the smear band regions including the expected sizes were excised. The library was recovered by incubating the shattered gel slice with 200 uL TE buffer overnight at 37 °C and the DNA in the supernatant was ethanol precipitated in presence of GlycoBlue (ThermoFisher Scientific). The multiplex 4C-seq library was pooled in equal molar ratio and sequenced on MiSeq (Illumina) with 2X250 bp or 1X150 bp. 500,000–1,000,000 reads were produced for each library. The viewpoint sequence was removed by Tagdust2 and the processed read was mapped by BOWTIE2 and over 80% of each library could be mapped to the hg19 genome. The mapped 4C-seq data was analysed by r3CSeq^30^.

### Chromatin Immunoprecipitation with quantitative PCR (ChIP-qPCR)

2 million cells were fixed with 1% formaldehyde in PBS for 10 minutes at room temperature. The fixation reaction was quenched by adding glycine to 0.25 M final concentration. The cell pellet was washed twice by cold PBS supplemented with 1 mM PMSF. The fixed cell was lysed in 100uL lysis buffer (Tris-HCl pH8, 10 mM EDTA pH8, 1% SDS, 1 mM PMSF) for 10 minutes on ice. The cell lysate were sonicated by Bioruptor (Diagenode) until 100 bp to 500 bp DNA fragment were obtained. The cell debris was cleared by spinning the sonicated lysate at 15000 RPM for 10 minutes at 4 °C. The lysate was diluted 10 times by dilution buffer (50 mM Tris-HCl pH8, 0.167 M NaCl, 1.1% TX-100, 0.11% sodium deoxycholate) and precleared by adding 100 uL of Protein A Dynabead (Thermo Scientific) and incubated at 4 °C for 2 hours. Antibody conjugated beads were prepared by adding 50 uL of antibody or IgG (total 1 ug in PBS/0.1% TX-100) to 50 uL Protein A Dynabeads in PBS/0.1% TX-100 and incubated at room temperature for 2 hours. The prepared antibody conjugated beads were added into 500 uL preclear lysate and incubated at 4 °C overnight. The beads were washed once with low salt washing buffer (50 mM Tris-HCl pH8, 0.15 M NaCl, 1 mM EDTA pH8, 0.1% SDS, 1% TX-100, 0.1% sodium deoxycholate), twice with high salt washing buffer (50 mM Tris-HCl pH8, 0.5 M NaCl, 1 mM EDTA pH8, 0.1% SDS, 1% TX-100, 0.1% sodium deoxycholate), twice with LiCl washing buffer (50 mM Tris-HCl pH8, 1 mM EDTA pH8, 1% IGEPAL CA630, 0.7% sodium deoxycholate, 0.5 M LiCl) and twice with TE buffer (10 mM Tris-HCl pH8, 1 mM EDTA pH8). The DNA was eluted and decrosslinked by incubating the bead with Elution Buffer (10 mM Tris-HCl pH8, 0.3 M NaCl, 5 mM EDTA pH8, 0.5% SDS) for 6 hours at 65 °C. The eluted DNA was purified by phenol-chloroform and precipitated with ethanol in the presence of glycogen. The ChIP-qPCR reactions were prepared using Gotaq qPCR Master Mix (Promega) and run on ABI 7500 Fast in duplicates. Antibodies used included normal rabbit IgG, sc-2027X from Santa Cruz and rabbit polyclonal H3K27ac ab4729 from Abcam. The primers used are listed in Table S8a.

### Patient sample collection

CML and non-CML patient samples were collected under Institute Review Board-approved procedures (National University Hospital of Singapore, DSRB-B/09/495) as part of the Cancer Science Institute Leukemia Cell Bank headed by Prof. Chng Wee Joo. Knee bone aspirate samples were collected under NHG Domain Specific Review Board-approved procedures (National University Hospital of Singapore, DSRB 2015/1037). Informed consent was obtained from all subjects.

Blood from completely normal individuals was not collected and stored as part of the bank. Blood from CML patients was collected. Blood from patients without CML (“non-CML”) but who had other diseases that were not expected to affect the blood was collected. Adult blood in the form of bone marrow (BM) and peripheral blood (PB) in EDTA was diluted in 1:1 ratio of Hank’s Balance Salt Solution (HBSS) or Phosphate Buffered Saline (PBS). Diluted BM and PB samples were layered on Ficoll paque medium in 1:1 ratio and centrifuged at 2000 RPM for 30 minutes with low deceleration. The mononuclear layer was collected and washed twice with 10 mL HBSS or PBS and centrifuged at 1500 RPM for 5 minutes. Upon the last wash, sample pellets were resuspended with cold freezing media (10% Dimethyl sulfoxide (DMSO), 90% Fetal Calf Serum (FCS)) in a ratio of 1 mL freezing media:10 million cells. Sample resuspension was performed in ice-water bath and all samples were subsequently transferred to cryovials and stored in liquid nitrogen for long-term storage.

Knee bone aspirate collected from normal individuals was resuspended in 50 ml PBS containing 10% FCS, 0.4% Sodium Citrate and 3 mM EDTA, and homogenized with a 5 ml syringe and 21 G needle. Homogenized samples were passed through a 100 μm cell strainer and pelleted at 300 × g for 10 minutes. Samples were then lysed in 50 ml lysis buffer (0.15 M Ammonium chloride, 10 mM Potassium bicarbonate, and 0.1 mM EDTA) following a 5-minute incubation at room temperature. After 5 minutes, blood cells were pelleted at 300 × g for 10 minutes at room temperature and resuspended in 13 ml PBS containing 2% FBS and 2 mM EDTA. Samples were then layered on Ficoll-Paque Plus in 6 ml cell suspension: 4 ml Ficoll ratio and centrifuged at 1000 × g for 20 minutes. Mononuclear layer was collected and washed twice with 10 mL PBS containing 2% FBS and 2 mM EDTA and once with PBS alone. Upon the last wash, knee BM samples were pelleted at 300 × g for 10 minutes and resuspended with cold freezing media (10% DMSO, 90% FCS) and transferred to cryovials, stored in liquid nitrogen for long-term storage. All patient sample information is listed in Table S5.

### EpiSwitch™

K562 cells were maintained for 1 week before 3–5 million cells were rinsed twice with PBS at room temperature and subsequently resuspended in PBS containing 10% glycerol, transferred to cryovials and stored in liquid nitrogen until shipping. Patient samples were directly shipped on dry ice to Oxford BioDynamics Plc.

All samples were processed for chromosome conformation capture analysis as per manufacturer’s instructions using EpiSwitch™ proprietary reagents and procedure developed for blood, tissue and cell lines analysis (Oxford BioDynamics Plc., Oxford, UK) as described previously^25, 26^. Briefly, following washes with PBS, cells were fixed with formaldehyde for 15 minutes and quenched with glycine. Cell lysis was performed and the nuclei was purified using density cushion centrifugation. TaqI and proximity ligation was performed using T4 DNA ligase, followed by subsequent proteinase K and RNAse A treatment to remove protein and RNA content. Upon DNA extraction from the samples, nested PCR was performed using primers listed in Table S8b.

All PCR amplified samples were then visualized by electrophoresis in the LabChip^”^ GX from Perkin Elmer, using the LabChip DNA 1 K Version2 kit (Perkin Elmer, Beaconsfield, UK) and internal DNA marker was loaded on the DNA chip according to the manufacturer’s protocol using fluorescent dyes. Fluorescence was detected by laser and electropherogram read-outs translated into a simulated band on gel picture using the instrument software. The threshold we set for a band to be deemed positive was 30 fluorescence units and above. The EpiSwitch™ bait and hit regions are described in Figure S8a. Additional information is provided in Figure S8b,c.

### RNA and DNA isolation

Total RNA and genomic DNA were isolated from patient blood samples using the AllPrep DNA/RNA/miRNA Universal Kit (Qiagen), incorporating on-column DNase and RNase digestions (Qiagen). Extracted RNA samples were analyzed on the Agilent 2100 bioanalyzer using the Agilent RNA 6000 Nano Kit (Agilent) while extracted DNA samples were separated on a 0.4% (w/v) 1x Tris/Acetate/EDTA buffered agarose gel to check for the quality of the extracted samples.

### Reverse Transcriptase Quantitative PCR (RT-qPCR)

cDNA was reverse transcribed from 500 ng total RNA extracted from the patient blood samples using the SuperScript III First-Strand Synthesis System using oligodT (Invitrogen). Quantitative real-time PCR (qPCR) was performed with 50 ng of reverse transcribed cDNA using the GoTaq qPCR Master Mix (Promega). All qPCR reactions were prepared in duplicates and analysed by the LightCycler 96 System (Roche) and quantitated using the Abs Quant/Fit Point analysis software (Roche). The mean expression levels of the threshold cycle number (Ct) of *c-MYC* were normalized by the Ct values of β-actin and the fold difference in expression levels was calculated using the 2^−ΔΔCt^ method. The primers used are listed in Table S8a.

### Sanger Sequencing

The Bait-Hit 1 region, Hotspot 1 region and Hotspot 2 region were amplified from extracted genomic DNA samples using the Phusion High-Fidelity PCR Master Mix (NEB) and sent for Sanger sequencing at Axil Scientific Pte Ltd. The raw data obtained was analysed using the Chromas Lite software v2.1.1 to look for mutations. The primers used are listed in Table S8a.

### Data Accession

The list of ChIP-Seq libraries used in the study were detailed in Supplementary Table 4. The 4C-seq libraries were deposited on GEO with accession GSE77125 (Reviewer link: https://www.ncbi.nlm.nih.gov/geo/query/acc.cgi?token=yfkjgggozfgpryv&acc=GSE77125).

### Declaration

All methods were carried out in accordance with relevant guidelines and regulations.

## Electronic supplementary material

Supplementary Materials

Supplementary Table 1

Supplementary Table 2

Supplementary Table 3

Supplementary Table 4
