# Supplementary material for: Super-Enhancers and Broad H3K4me3 Domains Form Complex Gene Regulatory Circuits Involving Chromatin Interactions
Source: Sci Rep. 2017 May 19;7:2186. doi: 10.1038/s41598-017-02257-3 (PMC5438348; doi:10.1038/s41598-017-02257-3)
Supplement: Supplementary file 1 — Supplementary Materials [file 41598_2017_2257_MOESM1_ESM.pdf]

Super-Enhancers and Broad H3K4me3 Domains Form Complex Gene Regulatory Circuits Involving Chromatin Interactions

Fan Cao[1*], Yiwen Fang[1*], Hong Kee Tan[1*], Yufen Goh[1], Jocelyn Yeen Hui Choy[1], Bryan Koh[2], Jiong Hao Tan[2], Nicolas Bertin[1,#], Aroul Ramadass[3], Ewan Hunter[3], Jayne Green[3], Matthew Salter[3], Alexandre Akoulitchev[3], Wilson Wang[2], Wee Joo Chng[1,2,7], Daniel G. Tenen[1], Melissa J. Fullwood[1, 4,5,6†]

[1]Cancer Science Institute of Singapore, National University of Singapore.
[2]National University Hospital, Singapore
[3]Oxford BioDynamics Plc, Oxford, England.
[4]School of Biological Sciences, Nanyang Technological University, Singapore.
[5]Institute of Molecular and Cell Biology, Agency for Science, Technology and Research (A*STAR), Singapore.
[6]Yale-NUS Liberal Arts College, Singapore.
[7] National University Cancer Institute, Singapore, National University Health System, Singapore

*These authors made equal and critical contributions
[#]Current address: Human Longevity Singapore Pte. Ltd., Singapore

[†]Correspondence should be sent to:
Melissa J. Fullwood
Cancer Science Institute Singapore (CSI),
National University of Singapore (NUS),
Centre for Translational Medicine (MD6),
14 Medical Drive, #12-01,
Singapore 117599.

Contents:

1. Supplementary Text

2. Supplementary Table Legends

3. Supplementary Figure Legends

4. Supplementary Tables

5. Supplementary Figures

Supplementary Text

EpiSwitch™ validation in K562

The EpiSwitch™ method was tested in K562 cells to check its efficacy and whether it could validate ChIA-PET data from K562 cells. Two technical repeats were screened for each region and 0/2 indicates no interaction observed while 1/2 and 2/2 indicates 1 and 2 interactions observed respectively in each individual K562 sample. For bait-hit2, there were 2 different primers designed at slightly different loci, indicated as bait-hit2-1 and bait-hit2-2. While there are a few differences, the results are mostly similar between bait-hit2-1 and bait-hit2-2. Negative control region 1, which has no reported interaction, was used for this analysis.

Individual heterogeneity in patients

We note that we also detected examples of individual heterogeneity in chromatin interactions in patients. While certain individuals had similar interaction patterns, others had different patterns – for example, *MYC* Bait-Hit1 was seen in 9 out of 13 peripheral blood samples examined while *MYC* Bait-Hit2-1 was seen in 12 out of 13 peripheral blood samples examined (**Figure S9a**). Interestingly, the *MYC* Bait-Hit1 interaction was observed in the peripheral blood but not bone marrow samples of patients AD454 and AD548 (**Figure S9a**). This particular interaction was also detected in only 4 out of 10 CML patient samples in contrast to 7 out of 8 non-CML patient samples that were screened **(Figure S9a)**.

Having observed heterogeneity between individuals in terms of chromatin interactions, we asked whether there was individual heterogeneity in enhancer activity levels. Due to limitations in the quantities of the patient samples, we were only able to examine two enhancers through the anti-H3K27ac ChIP-qPCR assay. We examined an enhancer at MYC-335, which is not present in K562 cells but is important in solid cancers, and the super-enhancer at *miR1205* which loops over to the *MYC* promoter in K562 cells. We found that the enhancer activity levels vary in individual samples (**Figure S9c**). We did not see any clear correlation between enhancer activity levels and chromatin interaction levels (**Table S5**).

Next, we investigated whether there were any genetic or transcriptomic differences between the patient samples, and if so, what might be the impact of genetic differences between samples on chromatin interactions and enhancer activity levels. We performed PCR amplification followed by capillary sequencing to determine the genotype of Single Nucleotide Polymorphisms as well as whether there were any novel somatic mutations in the region and at two cancer mutation hotspot regions [24] proximal of the *miR1205* super-enhancer in patient samples (**Table S4**). Although several SNPs varied between samples and several SNPs occurred in CTCF binding regions as measured by ChIP-Seq [25], none showed any correlation with chromatin interaction levels or enhancer levels. It should be noted, however, that no SNPs were found at the CTCF motif. We did not detect any different SNPs or mutations in the two mutation hotspot regions proximal of the *miR1205* super-enhancer. We did not detect by Sanger sequencing any novel somatic mutations in neither CML nor non-CML samples at the miR-1205 super-enhancer (**Table S4**). Taken together, we did not observe any clear correlations between different genetic status or the presence of novel DNA mutations and enhancer status or chromatin interaction levels or expression of *MYC* in the *miR1205* region examined (**Table S5**) in these clinical samples.

Supplementary Table Legends

Supplementary Table 1. Enhancers, Chromatin Interactions and Associated Genes (Excel file). Sheet 1 & 3, "All enhancers" indicates the entire set of super-enhancers and typical enhancers and whether they associate with any chromatin interactions, as well as their associated genes. Sheet 2 & 4, "Enhancers of both TSG and OG" indicates the entire set of enhancers that associate with tumor suppressor genes and oncogenes, either by proximity or through chromatin interactions. Column A-C: genomic coordinates of the enhancer element (A: chromosome; B: start; C: end). D: name of the element. E: signal level. F: strand. G: type of enhancer element - whether proximal or distal; whether a typical enhancer (TE) or super-enhancer (SE). H: gene transcripts which overlap with the enhancer element. The GENCODE transcript is reported, with the common gene name reported in brackets. Multiple transcripts are separated by a semicolon. Oncogenes are indicated by <OG>, tumor suppressors are indicated by <TSG>, and census cancer genes are indicated by <CCG>. If there is no gene, a dot is indicated in the cell instead. I: the CAGE expression levels of the genes indicated by the transcripts in H are reported here. J: the RNA-Seq expression levels of the genes indicated by the transcripts in H are reported here. K: number of interactions associated with the enhancer element. L: number of interacting distal super-enhancers (DSE). M: number of interacting proximal super-enhancers (PSE). N: number of interacting distal typical enhancers (DTE). O: number of interacting proximal typical enhancers (PTE). P: genes at proximal super-enhancers (PSE) that interact with this enhancer element. Q: the CAGE expression levels of the genes indicated by the transcripts in P are reported here. R: the RNA-Seq expression levels of the genes indicated by the transcripts in P are reported here. S: Genes at proximal typical enhancers (PTE) that interact with this enhancer element. T: the CAGE expression levels of the genes indicated by the transcripts in S are reported here. U: the RNA-Seq expression levels of the genes indicated by the transcripts in S are reported here. V: The maximum specificity score of the CAGE clusters at each transcript.

Supplementary Table 2. Listing of tumor suppressor genes, oncogenes and census cancer genes associated with super-enhancers and broad H3k4me3 domains (Excel file). This file lists all the tumor suppressor genes, oncogenes, and COSMIC census cancer genes that are targeted by super-enhancers or broad H3k4me3 domains by proximity or looping in K562 (Sheet 1) and MCF-7 (Sheet 2). Row 1 describes whether the genes are targeted by proximal super-enhancers (PSE)/broad H3k4me3 domains (PBD) or distal super-enhancers (DSE)/broad H3k4me3 domains (DBD) and whether they are targeted by proximity or by chromatin interactions.

Supplementary Table 3. H3k4me3 domains, Chromatin Interactions and Associated Genes (Excel file). Sheet 1 and 2 are data for K562 and MCF-7, respectively. Column A-C: genomic coordinates of the enhancer element (A: chromosome; B: start; C: end). D: name of the element. E: signal level. F: strand. G: type of enhancer element - whether proximal or distal; whether a typical domain (TD) or broad domain (BD). H: gene transcripts which overlap with the enhancer element. The GENCODE transcript is reported, with the common gene name reported in brackets. Multiple transcripts are separated by a semicolon. Oncogenes are indicated by <OG>, tumor suppressors are indicated by <TSG>, and census cancer genes are indicated by <CCG>. If there is no gene, a dot is indicated in the cell instead. I: the CAGE expression levels of the genes indicated by the transcripts in H are reported here. J: the RNA-Seq expression levels of the genes indicated by the transcripts in H are reported here. K: number of interactions associated with the enhancer element. L: number of interacting distal broad domains (DBD).

M: number of interacting proximal broad domains (PBD). N: number of interacting distal typical domains (DTD). O: number of interacting proximal typical domains (PTD). P: genes at proximal broad domains (PBD) that interact with this enhancer element. Q: the CAGE expression levels of the genes indicated by the transcripts in P are reported here. R: the RNA-Seq expression levels of the genes indicated by the transcripts in P are reported here. S: Genes at proximal typical domains (PTD) that interact with this enhancer element. T: the CAGE expression levels of the genes indicated by the transcripts in S are reported here. U: the RNA-Seq expression levels of the genes indicated by the transcripts in S are reported here. V: The maximum specificity score of the CAGE clusters at each transcript.

Supplementary Table 4. Listing of ChIP-Seq libraries used in the study.

Supplementary Table 5. Listing of patient samples examined (in this document). These samples include both peripheral blood and bone marrow from patients with chronic myelogenous leukemia as well as other diseases, and include knee bone aspirates from normal individuals.

Supplementary Table 6. Genetic variations in clinical samples (in this document). 6 SNPs were found in *miR1205* region. SNPs that fall within the CTCF regions are annotated with *. A>C indicates a change of base from A to C while A/C indicates two peaks identified from sequencing. No mutations and SNPs were found in the two DNA hotpsot regions that were sequenced. The genomic location for the *miR1205* sequenced region is chr8:128978404-128981253 while chr8:128,973,357-128,973,382 and chr8:129,066,969-129,067,005 are the genomic locations for DNA hotspot region 1 and 2 from Weinhold *et al.* respectively [1].

Supplementary Table 7. Cross-comparison between chromatin interactions, enhancers, gene expression data and sequencing at the miR1205 super-enhancer region (in this document). Enhancer activity is indicated by ChIP-qPCR of H3K27ac (**Figure S9C**). N.D. indicates "not done", due to low cell numbers in the clinical samples. Chromatin interactions are indicated as present (Yes) or absent (No) on the basis that 2 or 3 of the 3 tested interactions must be positive in order to conclude that the interaction is present. The source data is shown in **Figure S9A**. The *MYC* expression data is indicated by RT-qPCR (**Figure S9B**). N.D. indicates "not done", due to low cell numbers in the clinical samples. The source data of genetic signatures is from **Table S6**.

Supplementary Table 8. Listing of primers used in experiments (in this document). This is a listing of all primers used for ChIP-qPCR, RT-qPCR, DNA sequencing, and EpiSwitch^{TM}.

Supplementary Figure Legends

Figure S1. Schematic of analyses performed in this paper. The input data are indicated in light green, and the analyses performed are shown in light beige.

Figure S2. Characterizing proximal and distal super-enhancers. **a**. The signal profile of the super-enhancer calling and some oncogenes (red), tumor suppressor genes (blue), and census cancer genes (underlined) they target by proximity or by looping. b-c. Screenshots of super-enhancers at *FOXA1*, *GATA3* and *ESR1*, *CDKN1B*. **d**. Fraction of proximal elements found at leukaemia associated genes. **e.** Histone modifications (H3K27ac, H3K4me1, H3K4me3) at the four types of regulatory elements, including proximal super-enhancers (PSE), proximal typical enhancers (PTE), distal super-enhancers (DSE) and distal typical enhancers (DTE). **f-g**. The specificities **(f)** and expression levels **(g)** of CAGE clusters at proximal super-enhancers and proximal typical-enhancers. All boxplots presented were prepared in the following manner: the black horizontal line indicates the median, the top and bottom of the box indicates the third and first quartile respectively, and the whiskers indicate 1.5*the interquartile range. Widths of boxes are in proportion to the square root of the number of data points in each category and the statistics testing was done using Dunn's Test. **h-i**. The cell-type specificities and expressions of enhancer RNAs at distal super-enhancers (DSE) and distal typical enhancers (DTE). **j**. The number of distal enhancers, including distal super-enhancers (DSE) and distal typical enhancers (DTE) associated with enhancer transcription. TPM indicates tags per million sequences.

Figure S3. The overlap of enhancers with chromatin interactions and their effects on the transcription of the remote target genes **a**. Fractions of the four types of elements associated with at least one chromatin interaction. **b**. Boxplot for the number of interactions each type of element has (only interacting elements were included). **c**. Distribution of distances of the nearest TSS (blue), nearest active TSS (green), and TSSes through chromatin interactions (red) to the center of distal super-enhancers. **d-e**. The expression levels (**d**) and cell-type specificity scores (**e**) of CAGE clusters at proximal elements that are connected to the four types of elements as indicated on the x-axis. "None" indicates the set of CAGE clusters located at non-interacting proximal elements. (**f**) The expression levels of tumor suppressor genes (tsg), oncogenes (og), and cencus cancer genes (ccg) targeted by broad domains through proximity (pse_p) and looping (pse_d and dse_d) measured by RNA-Seq. Data shown is for MCF-7.

Figure S4. Analysis of broad H3K4me3 peaks and chromatin interactions. **a**. Rank of H3k4me3 peaks by size and the cancer associated genes targeted by some broad domains. **b**. Some histone modifications at the four types of H3k4me3 domains. **c.** The fraction of proximal broad and typical domains located near leukemia associated genes. p-value is produced Fisher's Exact Test. **d**. The fraction of each type of elements by H3k4me3 involved in chromatin interactions. **e-f**. The cell-type specificity scores **(e)** and expression levels **(f)** of CAGE clusters at proximal H3k4me3 elements that are connected to the four types of H3k4me3 elements as indicated on the x-axis. "None" indicates the set of CAGE clusters located at non-interacting proximal elements. **g**. The expression levels of tumor suppressor genes (tsg), oncogenes (og), and cencus cancer genes (ccg) targeted by broad domains through proximity (pbd_p) and looping (pbd_d and dbd_d) measured by RNA-Seq. Data shown is for MCF-7.

Figure S5. Normalization of the association between enhancers and chromatin interactions. **a-b**. Fraction of extended elements with chromatin interactions (**a**) and boxplot of number of chromatin interactions each type of elements has (**b**). **c.** Boxplot of number of chromatin interaction the four types of enhancer have after normalization against the total Pol2 signal present in the ChIA-PET dataset. **d-e**. Fraction of elements with chromatin interactions from HiC data (**d**) and boxplot of number of chromatin interactions from HiC data each type of elements has (**e**). **f-g.** Fraction of extended elements with chromatin interactions from HiC data (**f**) and boxplot of number of chromatin interactions from HiC data each type of elements has (**g**). The data shown is based on K562 cells.

Figure S6. Comparison of expressions (**a**) and specificities (**b**) of CAGE clusters at proximal super-enhancers/proximal typical enhancers involved in chromatin interactions and CAGE clusters at those not involved in chromatin interactions in K562 cells.

Figure S7. Overlap between super-enhancers and broad H3k4me3 domains in MCF-7 cells. **a.** Histone modifications at proximal regions with both super-enhancers and broad H3k4me3 domains (P_SE_BD), only super-enhancers (P_SE_O), only broad H3k4me3 domains (P_BD_O), and neither (P_O_O). **b.** Histone modifications at distal regions with both super-enhancers and broad H3k4me3 domains (D_SE_BD), super-enhancers only (D_SE_O), broad H3k4me3 domains only (D_BD_O), and neither (D_O_O). **c.** The fraction of different regions involved in chromatin interactions. **d.** Boxplot of number of chromatin interactions each type of regions is involved in. **e.** Pairwise comparison of enrichment at oncogenes (OG), tumor suppressor genes (TSG), COSMIC census cancer genes (CCG), and housekeeping genes (HKG) between different distal regions through looping (left), and different proximal regions through looping (middle) and proximity (right). Comparison was performed using Fisher's Exact Test followed by Holm-Šídák correction for multiple testing. The color indicates the product of negative log-transformed p-values and sign of the log-transformed corresponding odds ratio.

Figure S8. Characterization of chromatin interactions in human samples by EpiSwitch™. **a.** EpiSwitch™ tested regions were selected from analyzing K562 RNA Polymerase II ChIA-PET and super enhancer locations published in Hsniz et al. The *TP53* Bait region is located near the *TP53* promoter. The *TP53* Hit 1 region is located near the *MPDU1* gene promoter, and spans over *CD68*. The *TP53* Hit 2 region is located near the *KDM6B* gene promoter. All regions include proximal super-enhancers and proximal broad domains. The *MYC* Bait region is located at a proximal typical-enhancer, a proximal broad domain, and near the *MYC* promoter. The *MYC* Hit 1 region is located at a distal typical-enhancer; in a *PVT1* intron, and near *miR-1205*. *MYC* Hit 2 region is located at a distal super-enhancer, a distal typical domain; and in a *PVT1* intron. **b.** EpiSwitch™ results at the *MYC* locus. Results are shown at different input levels of DNA for semi-quantitative measurements, and with primers designed at each locus. Certain loci have two or more primer pairs used which indicate different locations within each locus. Results are shown from peripheral blood and bone marrow from Chronic Myelogenous Leukemia patients and control patients respectively. "1" indicates the interaction was detected, while "0" indicates the interaction was not detected. **c.** EpiSwitch™ results at the *TP53* locus. Results are shown at different input levels of DNA for semi-quantitative measurements, and with primers designed at each locus. Certain loci have two or more primer pairs used which indicate different locations within each locus. Results are shown from peripheral blood and bone marrow from Chronic Myelogenous Leukemia

patients and control patients respectively. “1” indicates the interaction was detected, while “0” indicates the interaction was not detected.

Figure S9. Characterization of chromatin interactions at *MYC* locus in human samples by EpiSwitch^{TM}, RT-qPCR and ChIP-qPCR. **a.** The interactions at the *MYC* locus were screened in triplicate and at multiple using 8 CML peripheral blood samples and 6 other patients (non-CML) peripheral blood. Two CML and two non-CML bone marrow samples were run as a pilot test of any possible differences in bone marrow (AD454 and AD548). “PB” indicates peripheral blood and “BM” indicates bone marrow. Three technical repeats were screened for each region and the number of interactions detected were indicated in numerical values out of the total three replicates for each of the individual patient samples. 2 or 3 of the 3 tested interactions must be positive to conclude that the interaction is detected. “*” indicates patient in blast crisis. It should be noted that this set of data was obtained in earlier experiments, before different input levels of DNA were used for semi-quantitative results. **b.** RT-qPCR comparison of *MYC* mRNA expression levels between CML and normal cells. *MYC* mRNA was quantified by qPCR and normalized against the expression of the β-actin housekeeping gene. The fold difference in *MYC* mRNA expression was then calculated relative to K562. Error bars indicate the mean + standard deviation. **c.** ChIP-qPCR of H3K27ac at MYC-335, a super-enhancer near miR-1205, *MYC* promoter and a negative control region. IgG showed low enrichment levels comparable to the negative control (results not shown). Error bars indicate the mean + standard deviation.

Figure S10. Analysis of regions with different super-enhancer and broad H3k4me3 domain coverage and their effects on transcription of target genes in K562. **a-b.** Counts of overlaps between H3k4me3 domains and H3k27ac enhancers. **c-d.** Specificities and expressions of CAGE clusters at different types proximal regions. **e-h.** The cell-type specificity scores (**e, g**) and expression levels (**f, h**) of CAGE clusters at proximal elements that are connected to regulatory regions as indicated on the x-axis. “None” indicates the set of CAGE clusters located at non-interacting proximal elements.

Figure S11. K562 RNA-Seq expression levels of oncogenes (OG), tumor suppressor genes (TSG), and census cancer genes (CCG) regulated by **a**. different types of distal regulatory regions through looping; **b**. different types of proximal regulatory regions through looping. **c**. different types of proximal regulatory regions by proximity.

Figure S12. Analysis of regions with different super-enhancer and broad H3k4me3 domain coverage and their effects on transcription of target genes in MCF-7. **a-b.** Counts of overlaps between H3k4me3 domains and H3k27ac enhancers. **c-d.** Specificities and expressions of CAGE clusters at different types proximal regions. **e-h.** The cell-type specificity scores (**e, g**) and expression levels (**f, h**) of CAGE clusters at proximal elements that are connected to regulatory regions as indicated on the x-axis. “None” indicates the set of CAGE clusters located at non-interacting proximal elements.

Figure S13. MCF-7 RNA-Seq expression levels of oncogenes (OG), tumor suppressor genes (TSG), and census cancer genes (CCG) regulated by **a**. different types of distal regulatory regions through looping; **b**. different types of proximal regulatory regions through looping. **c**. different types of proximal regulatory regions by proximity.

Figure S14. Enhancer sizes with enhancers ordered by the H3k27ac signals for K562 (**a**) and MCF-7 (**b**). A smooth window of 20 was applied. The vertical dotted line indicates the split between typical enhancers and super-enhancers.

Supplementary Tables

Sample ID	Sample type	Race	Gender	Age	Drug treatment	Description provided by clinic
**Chronic Myelogenous Leukemia (CML) samples**						
**AD548**	PB/BM	Asian	Female	40	Glivec/Hydroxyurea	CML – chronic
**AD454**	PB/BM	Asian	Male	38	Hydroxyurea	CML – accelerated
**AD35**	PB	Asian	Male	32	Hydroxyurea and IFN-alpha Glivec/Anagrelide	CML with blast transformation
**AD35a**	PB	Asian	Male	32	DAUNO/ARA-C 3 + 7 Glivec/Ara-C Anagrelide/Hydroxyurea	CML with blast transformation
**AD96**	PB	Asian	Female	34	Hydroxyurea/Glivec	CML – chronic
**AD256**	PB	Non-Asian	Male	43	Glivec	CML
**AD294**	PB	N/A	N/A	N/A	N/A	N/A
**AD301a**	PB	Asian	Female	41	Glivec	CML – chronic
**AD035**	PB	N/A	N/A	N/A	N/A	N/A
**AD096**	PB	Asian	Female	34	Imatini/Nilotinib	CML – chronic
**AD256**	PB	N/A	N/A	N/A	N/A	N/A
**AD264**	PB	Asian	Male	25	Imatinib	CML – chronic
**AD277**	PB	Asian	Male	29	Imatini/Nilotinib/Dasatinib	CML – chronic
**AD290a**	PB	Asian	Female	57	Imatinib	CML – chronic
**AD315**	PB	Asian	Male	64	Imatinib	CML – chronic
**AD60**	BM	Asian	Male	45	Hydroxyure/Glivec/HSCT	CML – chronic
**AD61**	BM	N/A	N/A	N/A	N/A	N/A
**AD166**	BM	N/A	N/A	N/A	N/A	N/A
**AD171**	BM	N/A	N/A	N/A	N/A	N/A
**AD173**	BM	Asian	Female	21	Hydroxyurea/Glivec/HSCT	CML – chronic
**AD202**	BM	Asian	Male	19	Nilotinib	CML – chronic
**AD206**	BM	Asian	Female	33	Nilotinib	CML – chronic
**AD212**	BM	Asian	Female	38	Glivec/Nilotinib	CML – chronic
**AD241**	BM	Asian	Male	31	Nilotinib	CML – chronic
**Non-Chronic Myelogenous Leukemia (non-CML) samples**						
**AD278**	PB	Asian	Male	42	N/A	Waldenstrom Macroglogulinemia
**AD283**	PB	Asian	Male	60	N/A	Splenic Marginal zone lymphomas
**AD289**	BM	Asian	Female	31	N/A	Reactive marrow

**AD339**	BM	Asian	Male	60	N/A	Clonal small B cell lymphoproliferative disease Mantle cell lymphoma excluded possibly atypical CLL.
**AD394**	PB	Asian	Male	55	N/A	No conclusive evidence of lymphoproliferative disease.
**AD412**	PB	Asian	Female	53	N/A	End stage renal failure
**AD419**	PB	N/A	N/A	N/A	N/A	N/A
**AD446**	PB	Asian	Female	33	N/A	Hodgkin Lymphoma
**AD272**	PB	Asian	Male	56	N/A	CEL with FIP1L1-PDGFRa positive
**AD276**	PB	Asian	Female	61	N/A	Hodgkin's lymphoma (IIIb)
**AD289**	PB	Asian	Female	32	N/A	Reactive marrow
**AD316**	PB	Asian	Male	69	N/A	Stage 4B T cell lymphoma
**AD334**	PB	Asian	Female	44	N/A	DLBCL
**AD346**	PB	Asian	Male	31	N/A	Hodgkin's disease
Normal Individuals (Knee Bone Aspirate samples)						
**Knee 8**	BM	Asian	Male	71	N/A	N/A
**Knee 9**	BM	Asian	Female	77	N/A	N/A
**Knee 10**	BM	Asian	Female	65	N/A	N/A
**Knee 11**	BM	Asian	Female	53	N/A	N/A
**Knee 12**	BM	Asian	Female	70	N/A	N/A
**Knee 13**	BM	Asian	Male	84	N/A	N/A
**Knee 14**	BM	Asian	Female	67	N/A	N/A
**Knee 15**	BM	Asian	Male	68	N/A	N/A

Table S5. Information on patient samples
*PB indicates Peripheral Blood; BM indicates Bone Marrow

**Patient Type**	**Sample ID**	**SNPs ID in *miR1205***					
		**rs755519**	**rs752235**	**rs890443**	**rs752427***	**rs752429***	**rs4733584***
**CML**	**AD256PB**	A/G	A/C	T>C	C/T	T/C	T/C
	**AD454PB**	A>G	A>C	T>C	C>T	T>C	T>C
	**AD301aPB**	A/G	A/C	T/C	C/T	T/C	T/C

	**AD548PB**	A>G	A>C	T>C	C>T	T>C	T>C
	**AD96PB**	A/G	A/C	T/C	C/T	T/C	T/C
	**AD294PB**	A>G	A>C	T>C	C>T	T>C	T>C
	**AD548BM**	A>G	A>C	T>C	C>T	T>C	T>C
	**AD35aPB**	A>G	A>C	T>C	C>T	T>C	T>C
	**AD35PB**	A>G	A>C	T>C	C>T	T>C	T>C
	**AD454BM**	A>G	A>C	T>C	C>T	T>C	T>C
**Non-CML**	**AD412PB**	A/G	A/C	T>C	C/T	T/C	T>C
	**AD289BM**	A>G	A>C	T>C	C>T	T>C	T>C
	**AD283PB**	A/G	A/C	T>C	C/T	T/C	T>C
	**AD339BM**	A>G	A>C	T>C	C>T	T>C	T>C

Table S6. Single nucleotide polymorphisms detected in the *miR1205* super-enhancer region.

**Sample ID**		**Enhancer Activity**	**Chromatin Interaction**	**MYC Gene Expression Level**	**Genetic Signatures**
**CML**	**AD35 PB**	17.13	No	0.37	rs755519 A>G<br>rs752235 A>C<br>rs890443 T>C<br>rs752427 C>T<br>rs752429 T>C<br>rs4733584 T>C
	**AD35a PB**	3.53	No	0.09	rs755519 A>G<br>rs752235 A>C<br>rs890443 T>C<br>rs752427 C>T<br>rs752429 T>C<br>rs4733584 T>C
	**AD96 PB**	2.98	Yes	N.D.	rs755519 A/G<br>rs752235 A/C<br>rs890443 T/C<br>rs752427 C/T<br>rs752429 T/C<br>rs4733584 T/C
	**AD256 PB**	N.D.	No	N.D.	rs755519 A/G<br>rs752235 A/C<br>rs890443 T>C

					rs752427 C/T<br>rs752429 T/C<br>rs4733584 T/C
	**AD294 PB**	N.D.	Yes	N.D.	rs755519 A>G<br>rs752235 A>C<br>rs890443 T>C<br>rs752427 C>T<br>rs752429 T>C<br>rs4733584 T>C
	**AD301a PB**	N.D.	No	N.D.	rs755519 A/G<br>rs752235 A/C<br>rs890443 T/C<br>rs752427 C/T<br>rs752429 T/C<br>rs4733584 T/C
	**AD454 PB**	N.D.	Yes	N.D.	rs755519 A>G<br>rs752235 A>C<br>rs890443 T>C<br>rs752427 C>T<br>rs752429 T>C<br>rs4733584 T>C
	**AD548 PB**	5.04	Yes	N.D.	rs755519 A>G<br>rs752235 A>C<br>rs890443 T>C<br>rs752427 C>T<br>rs752429 T>C<br>rs4733584 T>C
	**AD454 BM**	0.90	No	N.D.	rs755519 A>G<br>rs752235 A>C<br>rs890443 T>C<br>rs752427 C>T<br>rs752429 T>C<br>rs4733584 T>C
	**AD548 BM**	3.85	No	1.18	rs755519 A>G<br>rs752235 A>C<br>rs890443 T>C<br>rs752427 C>T<br>rs752429 T>C<br>rs4733584 T>C
**Non-CML**	**AD278 PB**	N.D.	Yes	N.D.	N.D.
	**AD283 PB**	2.64	Yes	0.33	rs755519 A/G<br>rs752235 A/C<br>rs890443 T>C<br>rs752427 C/T<br>rs752429 T/C<br>rs4733584 T>C
	**AD419 PB**	N.D.	Yes	N.D.	N.D.

	**AD446 PB**	N.D.	Yes	N.D.	N.D.
	**AD394 PB**	N.D.	No	N.D.	N.D.
	**AD412 PB**	10.0	Yes	N.D.	rs755519 A/G<br>rs752235 A/C<br>rs890443 T>C<br>rs752427 C/T<br>rs752429 T/C<br>rs4733584 T>C
	**AD289 BM**	N.D.	Yes	0.03	rs755519 A>G<br>rs752235 A>C<br>rs890443 T>C<br>rs752427 C>T<br>rs752429 T>C<br>rs4733584 T>C
	**AD339 BM**	7.68	Yes	N.D.	rs755519 A>G<br>rs752235 A>C<br>rs890443 T>C<br>rs752427 C>T<br>rs752429 T>C<br>rs4733584 T>C

Table S7. Cross-comparison between chromatin interactions, enhancers, gene expression data and sequencing.

Primer name	Sequence (5’ to 3’)
ChIP-qPCR primers	
MYC promoter Forward	AGAAGGGCAGGGCTTCTC
MYC promoter Reverse	CCGAAAACCGGCTTTTATAC
MYC-335 Forward	CAAAGGGCAGGAACCCAGCA
MYC-335 Reverse	GGCATGCCAAACCCTTCCCT
miR-1205 superenhancer Forward	GCTCCTGCCACAAAGACGGA
miR-1205 superenhancer Reverse	AAGGGAGCCTGTGTGTGCTC
Negative control Forward	GGCCAGAATCAGATTTTCCA
Negative Control Reverse	GCCTTAACTGAGCAGGCTGT
RT-qPCR primers	
MYC-Forward	TTCGGGTAGTGGAAAACCAG
MYC-Reverse	CAGCAGCTCGAATTTCTTCC
β-actin-Forward	ACCCTGAAGTACCCCATCGA
β-actin-Reverse	CTCAAACATGATCTGGGTCATCT
Genomic DNA amplification and Capillary Sequencing primers	
PVT1-1 Forward	TGCTGGTGGATCAATGTAGC
PVT1-1 Reverse	CACTGGCTACATAGGGAGGG
PVT1-2 Forward	GTGGGGCTGTTGAACACTTG
PVT1-2 Reverse	AGAAAGGCCCTGTCCTTCTG
PVT1-3 Forward	CGCTGATGACAAAGGGACAG
PVT1-3 Reverse	GGACCATCGTGAGCTCTTCT
PVT1-4 Forward	GCCACAAAGACGGAAAGGAA
PVT1-4 Reverse	AACAGACATCCCCAGACCTG
PVT1-5 Forward	AACTGCCTGGCTTCCTAACT
PVT1-5 Reverse	TCCCTTGACTTGGTGCTGTA
PVT1-6 Forward	TCTTGGGGTATGGTGAGCTC
PVT1-6 Reverse	TCAGGGTACAAATGGTCGCA
Hotspot 1 Forward	GGCACGAATAGGGGAGGAAT
Hotspot 1 Reverse	GGACCTCCCTCTGATCTTGT
Hotspot 2 Forward	CCACATGATTTGGGCACTCC
Hotspot 2 Reverse	AGTGACATCTGGACTGGTGT

4C Primers (Nested PCR)	
MYC 1st PCR Primer F	TGAAAGAATAACAAGGAGGTGGC
MYC 1st PCR Primer R	AGAAGGTCCGAAGAAAGAGGA
MYC 2nd PCR Primer 502	AATGATACGGCGACCACCGAGATCTACACCTCTCTATTCGTCGGCAGCGTCAGATGTGTATAAGAGACAGAACAAACCCTAAAACGGCCA
MYC 2nd PCR Primer 703	CAAGCAGAAGACGGCATACGAGATTTCTGCCTGTCTCGTGGGCTCGGAGATGTGTATAAGAGACAGACATCCAGGCGCGATGAT
MYC 2nd PCR Primer 704	CAAGCAGAAGACGGCATACGAGATGCTCAGGAGTCTCGTGGGCTCGGAGATGTGTATAAGAGACAGACATCCAGGCGCGATGAT
POLR2A 1st PCR Primer F	CCACTTAAGCACACAGACGG
POLR2A 1st PCR Primer R	TTGTCAGATGGCCCAGCTAA

Table S8a. Primer sequences used for ChIP-qPCR, RT-qPCR, Sanger Sequencing and 4C.

**EpiSwitch™ Primers (Nested PCR)**			
**Primer Name**		**Sequence**	**Description**
**MYC**	FULL2-19	CTCTCTCGCTAATCTCCGCC	Bait Forward Inner
	FULL2-20	TCATGCGGCTCTCTTACTCTG	Bait Forward Outer
	FULL2-23	ACTGGATCGGGGTAAAGTGAC	Bait Forward Inner
	FULL2-24	GCTGAGATGAGTCGAATGCC	Bait Forward Outer
	FULL2-25	TGTCCCTTTGTCATCAGCGG	Hit 1 Reverse Inner
	FULL2-26	AACACACCACTTGCTCAGGG	Hit 1 Reverse Outer
	FULL2-29	CGATGTTTTGAAGGACACAGTGG	Hit 2 Reverse Inner
	FULL2-30	AGCTCCGTCAGTGATCAGTC	Hit 2 Reverse Outer
	FULL2-33	GCATTCCTCTGGCTCCTCTG	Hit 2 Reverse Inner
	FULL2-34	GTCAAACTCCTTCCTGCAAGC	Hit 2 Reverse Outer
	FULL2-37	CTGAGAGTGAGCTGTGTGGG	Control Reverse Inner
	FULL2-38	TGCTACCTATTTCTGTCACCACC	Control Reverse Outer
**TP53**	FULL2-3	CGGTGCTAAGGAACACAGTG	Bait Forward Inner
	FULL2-4	CTAGG GCTTGATGGG AACGG	Bait Forward Outer
	FULL2-9	CAGGGAGGCTCATAACCTGG	Hit 2 Reverse Inner (Fig S7)
	FULL2-10	GGGTAGTGGTCAGCTGTAGG	Hit 2 Reverse Outer (Fig S7)
	FULL2-13	GCCTTTGTTGACGCTTCCAG	Hit 1 Reverse Inner
	FULL2-14	CTTTCTGACGCTACCCACCC	Hit 1 Reverse Outer
	Full2-41	TTATCCCTCTGTCCAGCGTC	Control Reverse Inner (Fig S7)
	Full2-42	GTTTCACGTCCCATGGTTGC	Control Reverse Outer (Fig S7)

	FULL2-5	TCCGGGGTGATTCTTTGCC	Control Reverse Inner (Fig S8)
	FULL2-6	ACCCATGAGCATCAGTCACC	Control Reverse Outer (Fig S8)
	FULL2-11	AAGAGGGATCACGGGTTTGG	Hit 2 Forward Inner (Fig S8)
	FULL2-12	AGAGGGTGGGGTTGACTGTC	Hit 2 Forward Outer (Fig S8)

Table S8b. Primer sequences used for EpiSwitch™.

Supplementary Figures

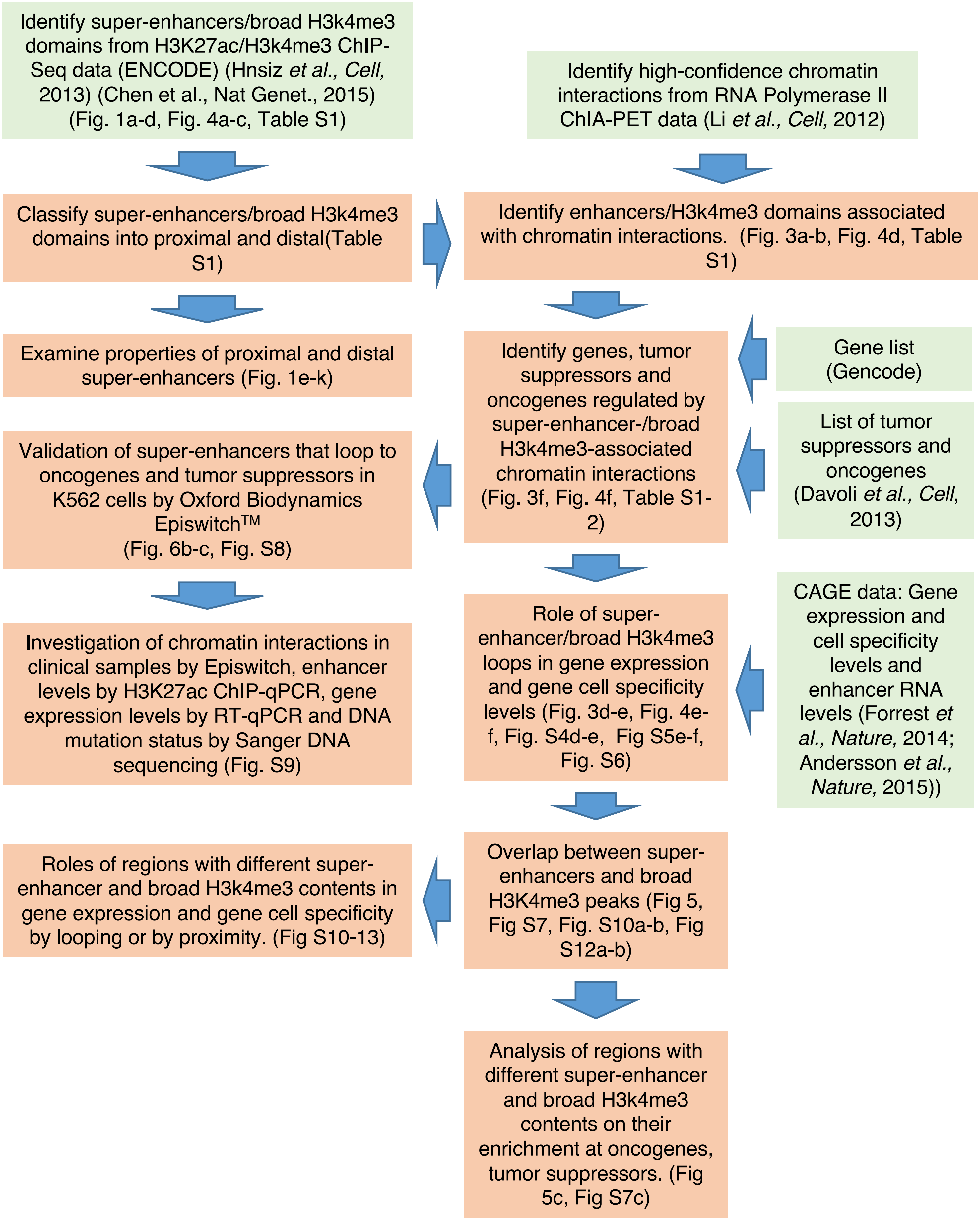
Identify super-enhancers/broad H3k4me3 domains from H3K27ac/H3k4me3 ChIP-Seq data (ENCODE) (Hnsiz *et al., Cell,* 2013) (Chen et al., Nat Genet., 2015) (Fig. 1a-d, Fig. 4a-c, Table S1)
Identify high-confidence chromatin interactions from RNA Polymerase II ChIA-PET data (Li *et al., Cell,* 2012)
Classify super-enhancers/broad H3k4me3 domains into proximal and distal(Table S1)
Identify enhancers/H3k4me3 domains associated with chromatin interactions. (Fig. 3a-b, Fig. 4d, Table S1)
Examine properties of proximal and distal super-enhancers (Fig. 1e-k)
Identify genes, tumor suppressors and oncogenes regulated by super-enhancer-/broad H3k4me3-associated chromatin interactions (Fig. 3f, Fig. 4f, Table S1-2)
Gene list (Gencode)
List of tumor suppressors and oncogenes (Davoli *et al., Cell*, 2013)
Validation of super-enhancers that loop to oncogenes and tumor suppressors in K562 cells by Oxford Biodynamics Episwitch™ (Fig. 6b-c, Fig. S8)
Investigation of chromatin interactions in clinical samples by Episwitch, enhancer levels by H3K27ac ChIP-qPCR, gene expression levels by RT-qPCR and DNA mutation status by Sanger DNA sequencing (Fig. S9)
Role of super-enhancer/broad H3k4me3 loops in gene expression and gene cell specificity levels (Fig. 3d-e, Fig. 4e-f, Fig. S4d-e, Fig S5e-f, Fig. S6)
CAGE data: Gene expression and cell specificity levels and enhancer RNA levels (Forrest *et al., Nature,* 2014; Andersson *et al., Nature,* 2015))
Roles of regions with different super-enhancer and broad H3k4me3 contents in gene expression and gene cell specificity by looping or by proximity. (Fig S10-13)
Overlap between super-enhancers and broad H3K4me3 peaks (Fig 5, Fig S7, Fig. S10a-b, Fig S12a-b)
Analysis of regions with different super-enhancer and broad H3k4me3 contents on their enrichment at oncogenes, tumor suppressors. (Fig 5c, Fig S7c)

Figure S1

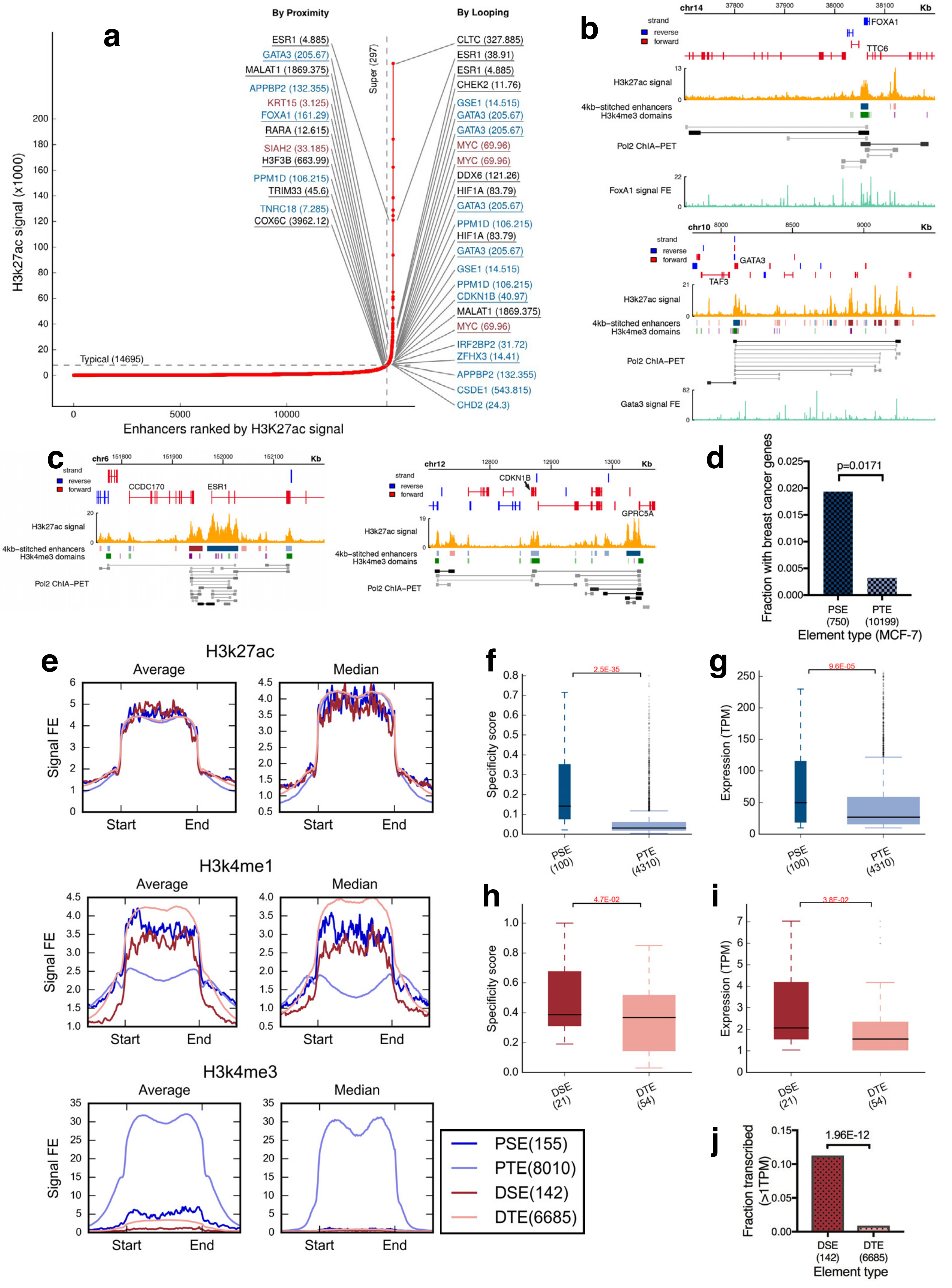

a
By Proximity
By Looping
Super (297)
Typical (14695)
H3k27ac signal (x1000)
Enhancers ranked by H3K27ac signal
ESR1 (4.885)
GATA3 (205.67)
MALAT1 (1869.375)
APPBP2 (132.355)
KRT15 (3.125)
FOXA1 (161.29)
RARA (12.615)
SIAH2 (33.185)
H3F3B (663.99)
PPM1D (106.215)
TRIM33 (45.6)
TNRC18 (7.285)
COX6C (3962.12)
CLTC (327.885)
ESR1 (38.91)
ESR1 (4.885)
CHEK2 (11.76)
GSE1 (14.515)
GATA3 (205.67)
GATA3 (205.67)
MYC (69.96)
MYC (69.96)
DDX6 (121.26)
HIF1A (83.79)
GATA3 (205.67)
PPM1D (106.215)
HIF1A (83.79)
GATA3 (205.67)
GSE1 (14.515)
PPM1D (106.215)
CDKN1B (40.97)
MALAT1 (1869.375)
MYC (69.96)
IRF2BP2 (31.72)
ZFHX3 (14.41)
APPBP2 (132.355)
CSDE1 (543.815)
CHD2 (24.3)
b
chr14
strand
reverse
forward
FOXA1
TTC6
H3k27ac signal
4kb-stitched enhancers
H3k4me3 domains
Pol2 ChIA-PET
FoxA1 signal FE
chr10
GATA3
TAF3
Gata3 signal FE
Kb
c
chr6
CCDC170
ESR1
chr12
CDKN1B
GPRC5A
d
p=0.0171
Fraction with breast cancer genes
PSE (750)
PTE (10199)
Element type (MCF-7)
e
H3k27ac
H3k4me1
H3k4me3
Average
Median
Signal FE
Start
End
PSE(155)
PTE(8010)
DSE(142)
DTE(6685)
f
2.5E-35
Specificity score
PSE (100)
PTE (4310)
g
9.6E-05
Expression (TPM)
h
4.7E-02
DSE (21)
DTE (54)
i
3.8E-02
j
1.96E-12
Fraction transcribed (>1TPM)
DSE (142)
DTE (6685)
Element type

Figure S2

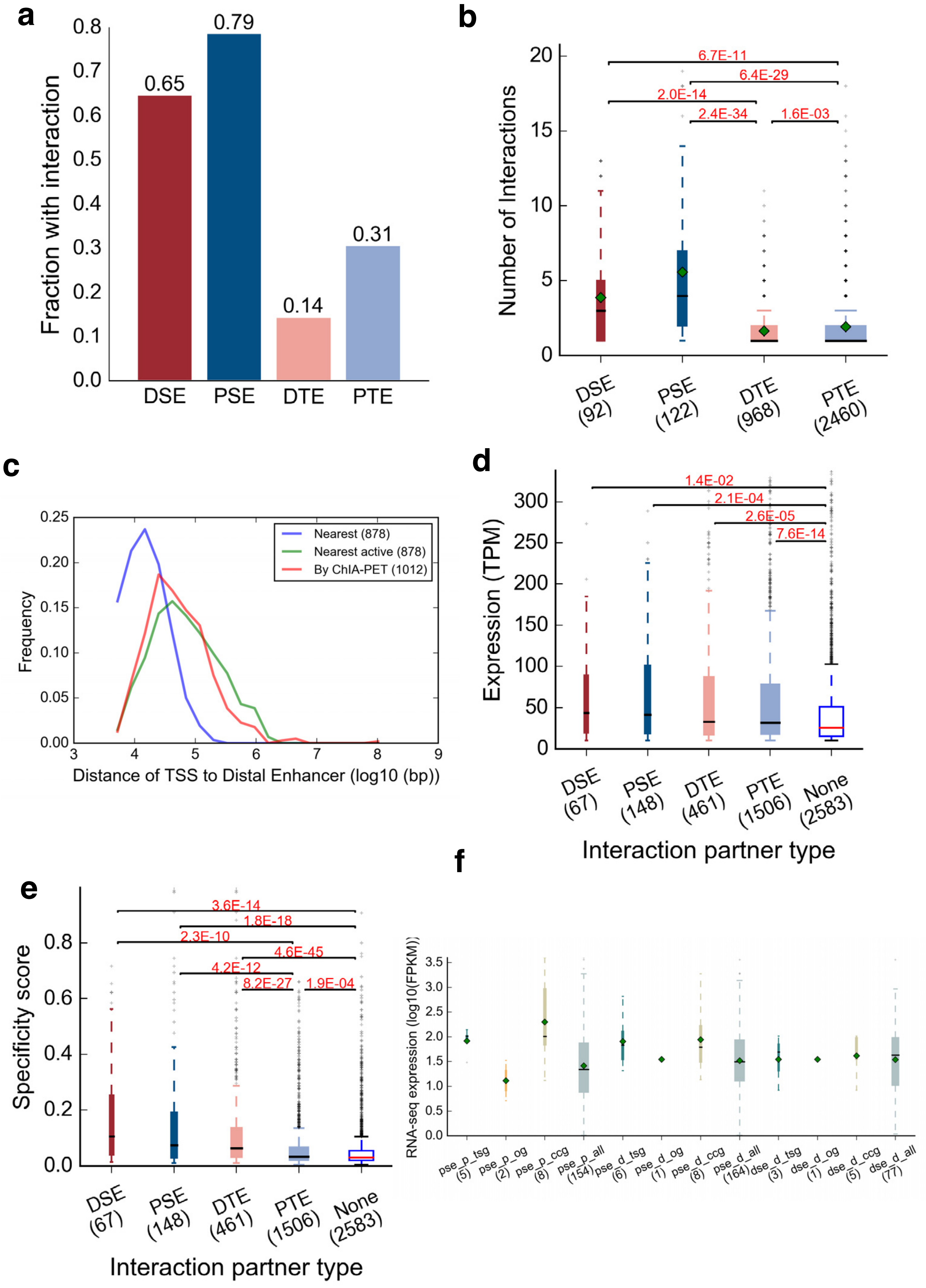
a
Fraction with interaction
0.0
0.1
0.2
0.3
0.4
0.5
0.6
0.7
0.8
0.65
0.79
0.14
0.31
DSE
PSE
DTE
PTE
b
Number of Interactions
0
5
10
15
20
6.7E-11
6.4E-29
2.0E-14
2.4E-34
1.6E-03
DSE (92)
PSE (122)
DTE (968)
PTE (2460)
c
Frequency
Nearest (878)
Nearest active (878)
By ChIA-PET (1012)
Distance of TSS to Distal Enhancer (log10 (bp))
d
Expression (TPM)
1.4E-02
2.1E-04
2.6E-05
7.6E-14
DSE (67)
PSE (148)
DTE (461)
PTE (1506)
None (2583)
Interaction partner type
e
Specificity score
3.6E-14
1.8E-18
2.3E-10
4.6E-45
4.2E-12
8.2E-27
1.9E-04
DSE (67)
PSE (148)
DTE (461)
PTE (1506)
None (2583)
Interaction partner type
f
RNA-seq expression (log10(FPKM))
pse_p_tsg (5)
pse_p_og (2)
pse_p_ccg (8)
pse_p_all (154)
pse_d_tsg (6)
pse_d_og (1)
pse_d_ccg (8)
pse_d_all (164)
dse_d_tsg (3)
dse_d_og (1)
dse_d_ccg (5)
dse_d_all (77)

Figure S3

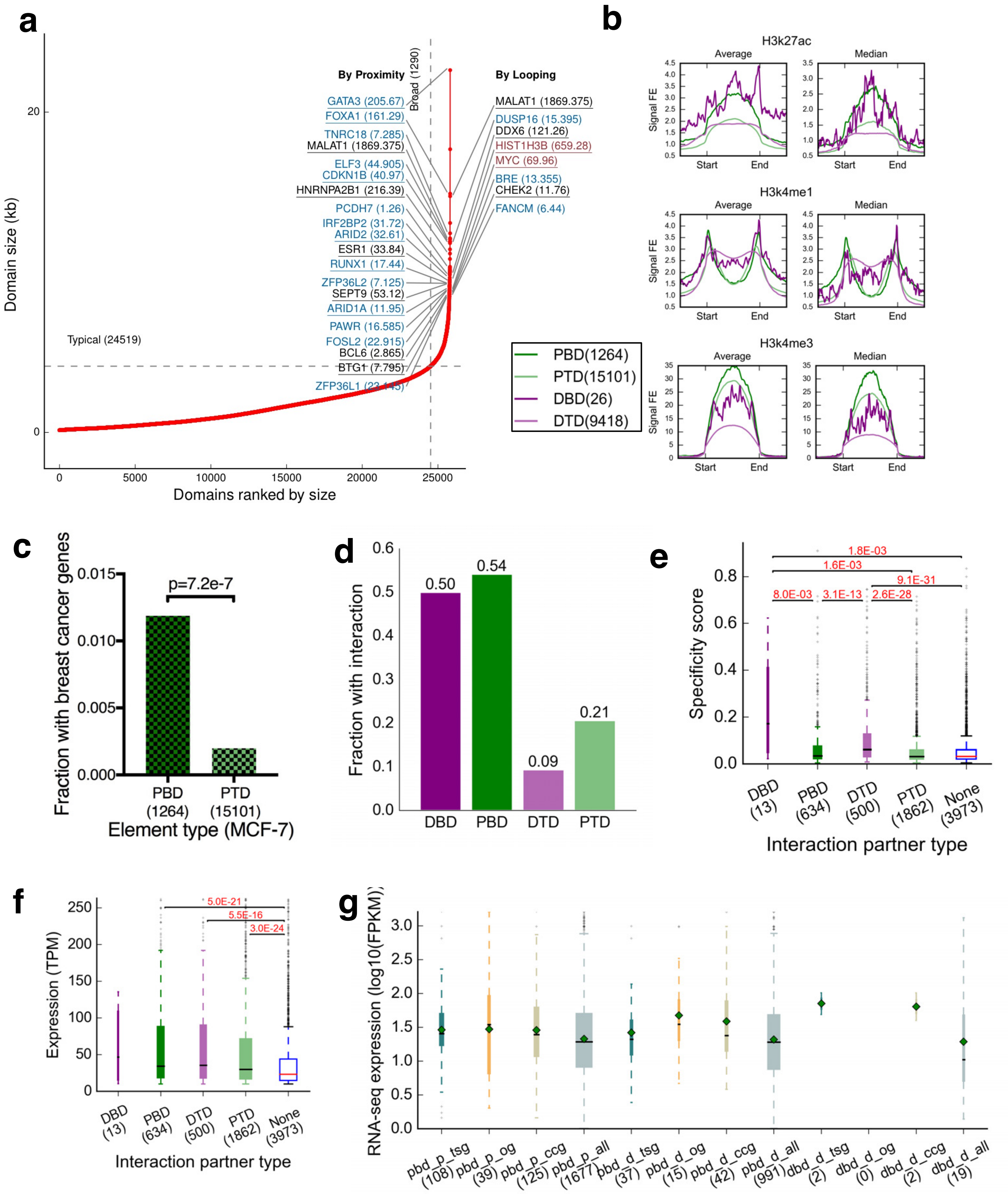
a
By Proximity
By Looping
Broad (1290)
Typical (24519)
GATA3 (205.67)
FOXA1 (161.29)
TNRC18 (7.285)
MALAT1 (1869.375)
ELF3 (44.905)
CDKN1B (40.97)
HNRNPA2B1 (216.39)
PCDH7 (1.26)
IRF2BP2 (31.72)
ARID2 (32.61)
ESR1 (33.84)
RUNX1 (17.44)
ZFP36L2 (7.125)
SEPT9 (53.12)
ARID1A (11.95)
PAWR (16.585)
FOSL2 (22.915)
BCL6 (2.865)
BTG1 (7.795)
MALAT1 (1869.375)
DUSP16 (15.395)
DDX6 (121.26)
HIST1H3B (659.28)
MYC (69.96)
BRE (13.355)
CHEK2 (11.76)
FANCM (6.44)
Domain size (kb)
Domains ranked by size
b
H3k27ac
H3k4me1
H3k4me3
Average
Median
Signal FE
Start
End
PBD(1264)
PTD(15101)
DBD(26)
DTD(9418)
c
p=7.2e-7
Fraction with breast cancer genes
PBD (1264)
PTD (15101)
Element type (MCF-7)
d
Fraction with interaction
0.50
0.54
0.09
0.21
DBD
PBD
DTD
PTD
e
Specificity score
1.8E-03
1.6E-03
9.1E-31
8.0E-03
3.1E-13
2.6E-28
DBD (13)
PBD (634)
DTD (500)
PTD (1862)
None (3973)
Interaction partner type
f
Expression (TPM)
5.0E-21
5.5E-16
3.0E-24
DBD (13)
PBD (634)
DTD (500)
PTD (1862)
None (3973)
Interaction partner type
g
RNA-seq expression (log10(FPKM))
pbd_p_tsg (108)
pbd_p_og (39)
pbd_p_ccg (125)
pbd_p_all (1677)
pbd_d_tsg (37)
pbd_d_og (15)
pbd_d_ccg (42)
pbd_d_all (991)
dbd_d_tsg (2)
dbd_d_og (0)
dbd_d_ccg (2)
dbd_d_all (19)

Figure S4

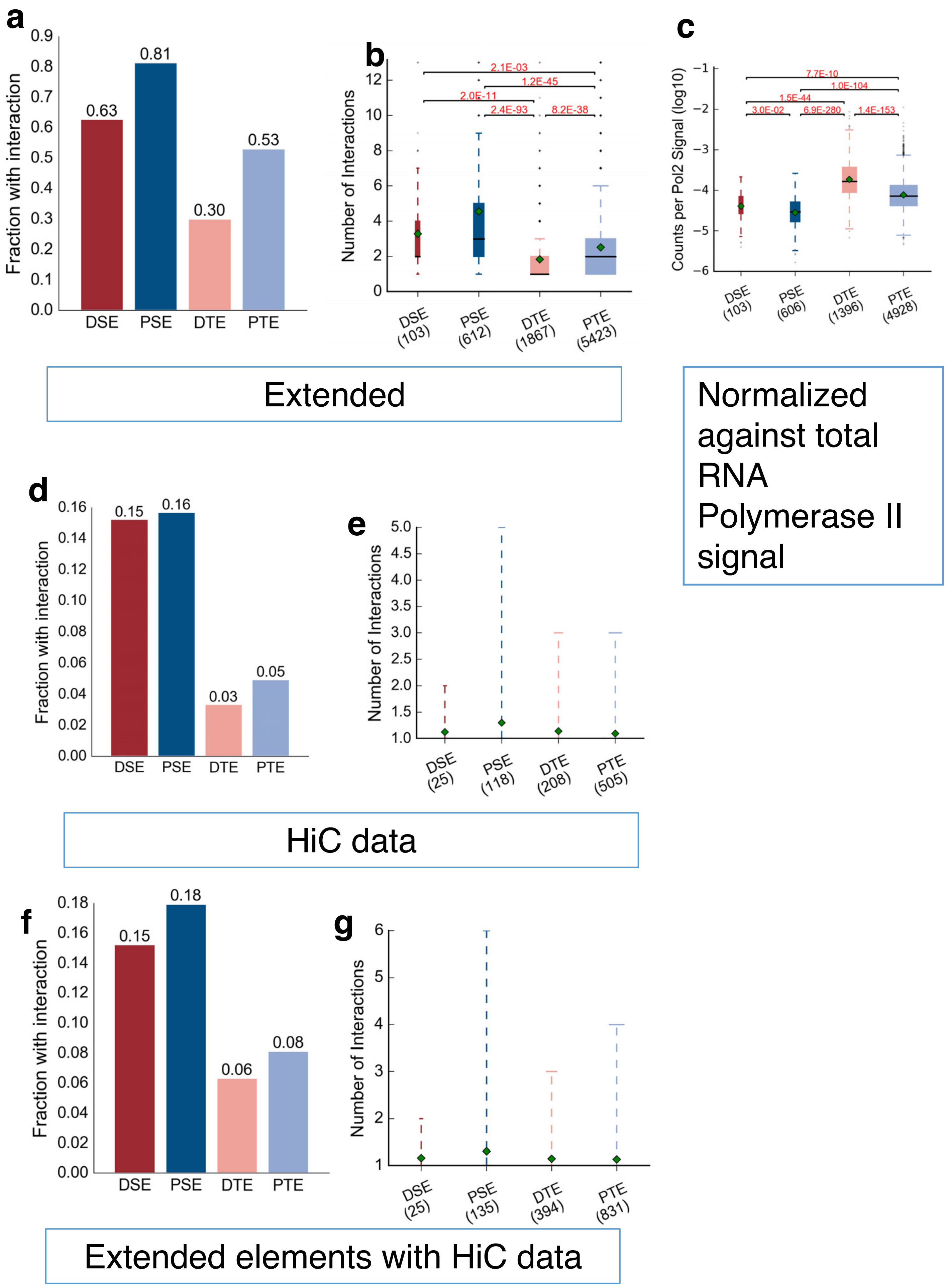
a
Fraction with interaction
0.9
0.8
0.7
0.6
0.5
0.4
0.3
0.2
0.1
0.0
0.63
0.81
0.30
0.53
DSE PSE DTE PTE
b
Number of Interactions
0
2
4
6
8
10
12
2.1E-03
1.2E-45
2.0E-11
2.4E-93
8.2E-38
DSE (103)
PSE (612)
DTE (1867)
PTE (5423)
c
Counts per Pol2 Signal (log10)
−1
−2
−3
−4
−5
−6
7.7E-10
1.0E-104
1.5E-44
3.0E-02
6.9E-280
1.4E-153
DSE (103)
PSE (606)
DTE (1396)
PTE (4928)
Extended
Normalized against total RNA Polymerase II signal
d
Fraction with interaction
0.16
0.14
0.12
0.10
0.08
0.06
0.04
0.02
0.00
0.15
0.16
0.03
0.05
DSE PSE DTE PTE
e
Number of Interactions
5.0
4.5
4.0
3.5
3.0
2.5
2.0
1.5
1.0
DSE (25)
PSE (118)
DTE (208)
PTE (505)
HiC data
f
Fraction with interaction
0.18
0.16
0.14
0.12
0.10
0.08
0.06
0.04
0.02
0.00
0.15
0.18
0.06
0.08
DSE PSE DTE PTE
g
Number of Interactions
6
5
4
3
2
1
DSE (25)
PSE (135)
DTE (394)
PTE (831)
Extended elements with HiC data

Figure S5

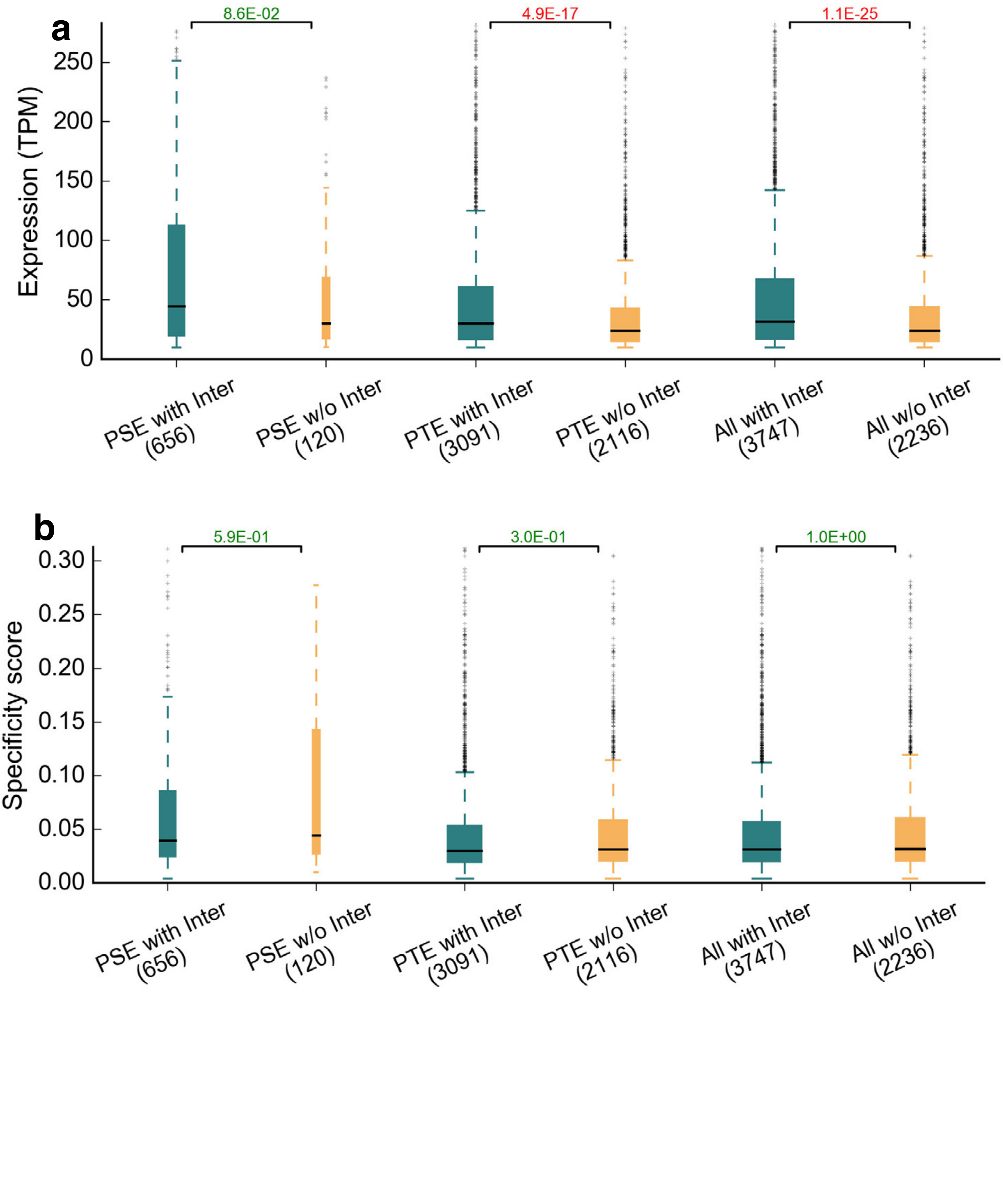
a
8.6E-02
4.9E-17
1.1E-25
Expression (TPM)
0
50
100
150
200
250
PSE with Inter (656)
PSE w/o Inter (120)
PTE with Inter (3091)
PTE w/o Inter (2116)
All with Inter (3747)
All w/o Inter (2236)
b
5.9E-01
3.0E-01
1.0E+00
Specificity score
0.00
0.05
0.10
0.15
0.20
0.25
0.30
PSE with Inter (656)
PSE w/o Inter (120)
PTE with Inter (3091)
PTE w/o Inter (2116)
All with Inter (3747)
All w/o Inter (2236)

Figure S6

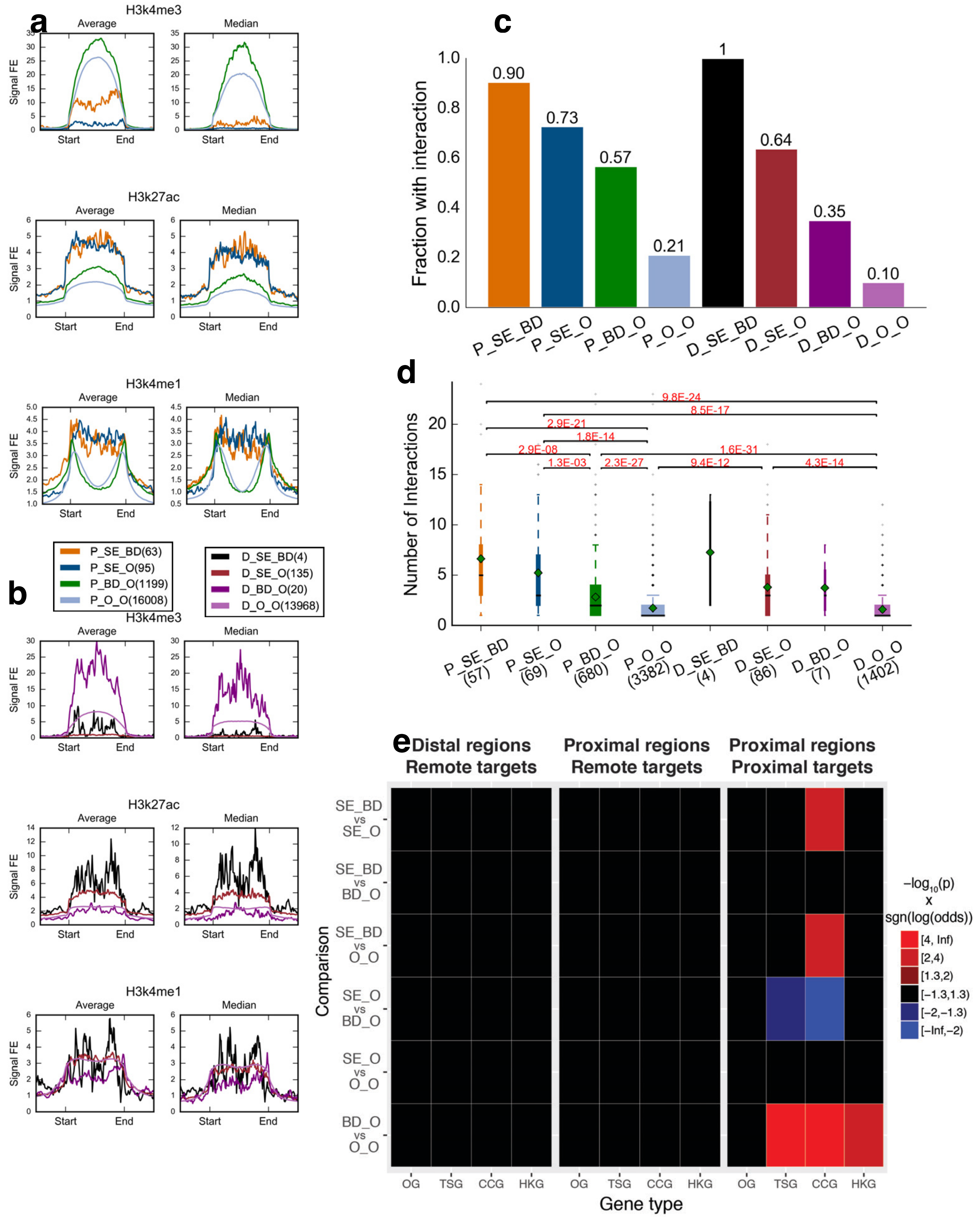

a
H3k4me3
Average
Median
Signal FE
Start
End
H3k27ac
H3k4me1
P_SE_BD(63)
P_SE_O(95)
P_BD_O(1199)
P_O_O(16008)
D_SE_BD(4)
D_SE_O(135)
D_BD_O(20)
D_O_O(13968)
b
c
Fraction with interaction
0.90
0.73
0.57
0.21
1
0.64
0.35
0.10
P_SE_BD
P_SE_O
P_BD_O
P_O_O
D_SE_BD
D_SE_O
D_BD_O
D_O_O
d
Number of Interactions
9.8E-24
8.5E-17
2.9E-21
1.8E-14
2.9E-08
1.6E-31
1.3E-03
2.3E-27
9.4E-12
4.3E-14
P_SE_BD (57)
P_SE_O (69)
P_BD_O (680)
P_O_O (3382)
D_SE_BD (4)
D_SE_O (86)
D_BD_O (7)
D_O_O (1402)
e
Distal regions Remote targets
Proximal regions Remote targets
Proximal regions Proximal targets
Comparison
SE_BD vs SE_O
SE_BD vs BD_O
SE_BD vs O_O
SE_O vs BD_O
SE_O vs O_O
BD_O vs O_O
OG
TSG
CCG
HKG
Gene type
−log10(p) x sgn(log(odds))
[4, Inf)
[2,4)
[1.3,2)
[−1.3,1.3)
[−2,−1.3)
[−Inf,−2)

Figure S7

a

EpiSwitch™ regions at *c-MYC*	Characteristics	EpiSwitch™ regions at *TP53*	Characteristics
***c-MYC* Bait**	Proximal typical-enhancer; proximal broad domain; near the *c-MYC* promoter	***TP53* Bait**	Proximal super-enhancer; proximal broad domain; near the *TP53* promoter
***c-MYC* Hit 1**	Distal typical-enhancer; in a *PVT1* intron; near *miR-1205*	***TP53* Hit 1**	Proximal super-enhancer; proximal broad domain; near the *MPDU1* gene promoter; spanning over *CD68*
***c-MYC* Hit 2**	Distal super-enhancer; distal typical domain; in a *PVT1* intron	***TP53* Hit 2**	Proximal super-enhancer; proximal broad domain; near the *KDM6B* gene promoter
**Negative control**	No genes; quiescent region	**Negative control**	In a *DNAH2* intron; quiescent region

b

				Quality controlled Sample Data																																Detection rates (%)						
				CML (Bone Marrow)									Control (Bone Marrow)								CML (Peripheral Blood)									Control (Peripheral Blood)												
Genomic Loci	Interaction (F/R)	Primer Set and Dilution	DNA Input (ng)	AD60	AD61	AD166	AD171	AD173	AD202	AD206	AD212	AD241	Knee 8	Knee 9	Knee 10	Knee 11	Knee 12	Knee 13	Knee 14	Knee 15	AD035	AD096	AD256	AD264	AD277	AD290a	AD294	AD301a	AD315	AD272	AD276	AD289	AD316	AD334	AD346	Sample detection (%)	CML BM (%)	Control BM (%)	CML PB (%)	Control PB (%)	Total CML (%)	Total Control (%)
c-MYC	Bait (1)-Hit 1	FULL2 19/25_1X	13	0	0	0	0	0	0	0	0	0	0	0	0	0	0	1	0	0	1	0	0	0	0	0	0	0	0	0	0	1	1	0	0	12.5	0	13	11	33	5.6	21
		FULL2 19/25_1/2X	6.5	0	0	0	0	0	0	0	0	0	0	0	0	0	0	0	0	0	1	0	0	0	0	0	0	0	0	0	0	1	1	0	0	9.38	0	0	11	33	5.6	14
		FULL2 19/25_1/4X	3.25	0	0	0	0	0	0	0	0	0	0	0	0	0	0	0	0	0	0	0	0	0	0	0	0	0	0	0	0	0	1	0	0	3.13	0	0	0	17	0	7.1
		FULL2 19/25_1/8X	1.625	0	0	0	0	0	0	0	0	0	0	0	0	0	0	0	0	0	0	0	0	0	0	0	0	0	0	0	0	0	0	0	0	0	0	0	0	0	0	0
		FULL2 19/25_1/16X	0.8125	0	0	0	0	0	0	0	0	0	0	0	0	0	0	0	0	0	0	0	0	0	0	0	0	0	0	0	0	0	0	0	0	0	0	0	0	0	0	0
		FULL2 19/25_1/32X	0.40625	0	0	0	0	0	0	0	0	0	0	0	0	0	0	0	0	0	0	0	0	0	0	0	0	0	0	0	0	0	0	0	0	0	0	0	0	0	0	0
	Bait (2)-Hit 1	FULL2 23/25_1X	13	0	0	1	0	1	0	0	0	0	1	1	0	0	0	0	0	1	0	0	0	0	0	0	0	0	0	0	0	0	1	1	0	21.9	22	38	0	33	11	36
		FULL2 23/25_1/2X	6.5	0	0	0	0	0	0	0	0	0	1	0	0	0	0	0	0	1	0	0	0	0	0	0	0	0	0	0	0	0	1	1	0	12.5	0	25	0	33	0	29
		FULL2 23/25_1/4X	3.25	0	0	0	0	0	0	0	0	0	0	0	0	0	0	0	0	0	0	0	0	0	0	0	0	0	0	0	0	0	0	0	0	0	0	0	0	0	0	0
		FULL2 23/25_1/8X	1.625	0	0	0	0	0	0	0	0	0	0	0	0	0	0	0	0	0	0	0	0	0	0	0	0	0	0	0	0	0	0	0	0	0	0	0	0	0	0	0
		FULL2 23/25_1/16X	0.8125	0	0	0	0	0	0	0	0	0	0	0	0	0	0	0	0	0	0	0	0	0	0	0	0	0	0	0	0	0	0	0	0	0	0	0	0	0	0	0
		FULL2 23/25_1/32X	0.40625	0	0	0	0	0	0	0	0	0	0	0	0	0	0	0	0	0	0	0	0	0	0	0	0	0	0	0	0	0	0	0	0	0	0	0	0	0	0	0
	Bait (1)-Hit 2	FULL2 19/29_1X	13	1	0	0	0	0	0	1	0	0	1	0	1	1	1	0	1	1	1	1	1	1	0	1	1	1	0	1	1	1	1	0	1	62.5	22	75	78	83	50	79
		FULL2 19/29_1/2X	6.5	1	0	0	0	0	0	1	0	0	1	0	1	1	1	0	1	1	0	0	0	1	0	0	1	1	0	1	0	0	1	0	1	43.8	22	75	33	50	28	64
		FULL2 19/29_1/4X	3.25	0	0	0	0	0	0	0	0	0	0	0	1	0	0	0	0	0	0	0	0	0	0	0	1	1	0	0	0	0	0	0	1	12.5	0	13	22	17	11	14
		FULL2 19/29_1/8X	1.625	0	0	0	0	0	0	0	0	0	0	0	0	0	0	0	0	0	0	0	0	0	0	0	0	0	0	0	0	0	0	0	0	0	0	0	0	0	0	0
		FULL2 19/29_1/16X	0.8125	0	0	0	0	0	0	0	0	0	0	0	0	0	0	0	0	0	0	0	0	0	0	0	0	0	0	0	0	0	0	0	0	0	0	0	0	0	0	0
		FULL2 19/29_1/32X	0.40625	0	0	0	0	0	0	0	0	0	0	0	0	0	0	0	0	0	0	0	0	0	0	0	0	0	0	0	0	0	0	0	0	0	0	0	0	0	0	0
		FULL2 19/33_1X	13	1	1	1	1	1	1	0	0	0	1	1	1	1	0	1	1	1	1	1	0	1	0	1	1	1	0	1	0	0	1	0	1	68.8	67	88	67	50	67	71
		FULL2 19/33_1/2X	6.5	0	1	1	0	0	1	0	0	0	1	1	1	1	0	1	1	0	0	1	0	1	0	0	1	1	0	1	0	0	1	0	0	46.9	33	75	44	33	39	57
		FULL2 19/33_1/4X	3.25	0	0	0	0	0	0	0	0	0	1	0	1	1	0	1	0	0	0	1	0	1	0	0	1	1	0	0	0	0	0	0	0	25	0	50	44	0	22	29
		FULL2 19/33_1/8X	1.625	0	0	0	0	0	0	0	0	0	1	0	0	0	0	0	0	0	0	1	0	1	0	0	1	0	0	0	0	0	0	0	0	12.5	0	13	33	0	17	7.1
		FULL2 19/33_1/16X	0.8125	0	0	0	0	0	0	0	0	0	0	0	0	0	0	0	0	0	0	0	0	0	0	0	0	0	0	0	0	0	0	0	0	0	0	0	0	0	0	0
		FULL2 19/33_1/32X	0.40625	0	0	0	0	0	0	0	0	0	0	0	0	0	0	0	0	0	0	0	0	0	0	0	0	0	0	0	0	0	0	0	0	0	0	0	0	0	0	0
	Bait (2) - Hit 2	FULL2 23/29_1X	13	0	0	0	0	0	0	0	0	0	1	0	1	0	0	1	1	0	0	1	1	0	0	0	0	0	0	0	1	1	0	0	1	28.1	0	50	22	50	11	50
		FULL2 23/29_1/2X	6.5	0	0	0	0	0	0	0	0	0	0	0	1	0	0	1	1	0	0	1	0	0	0	0	0	0	0	0	0	0	0	0	0	12.5	0	38	11	0	5.6	21
		FULL2 23/29_1/4X	3.25	0	0	0	0	0	0	0	0	0	0	0	0	0	0	1	0	0	0	0	0	0	0	0	0	0	0	0	0	0	0	0	0	3.13	0	13	0	0	0	7.1
		FULL2 23/29_1/8X	1.625	0	0	0	0	0	0	0	0	0	0	0	0	0	0	0	0	0	0	0	0	0	0	0	0	0	0	0	0	0	0	0	0	0	0	0	0	0	0	0
		FULL2 23/29_1/16X	0.8125	0	0	0	0	0	0	0	0	0	0	0	0	0	0	0	0	0	0	0	0	0	0	0	0	0	0	0	0	0	0	0	0	0	0	0	0	0	0	0
		FULL2 23/29_1/32X	0.40625	0	0	0	0	0	0	0	0	0	0	0	0	0	0	0	0	0	0	0	0	0	0	0	0	0	0	0	0	0	0	0	0	0	0	0	0	0	0	0
		FULL2 23/33_1X	13	0	0	0	1	0	0	0	0	1	0	0	0	0	0	1	0	0	1	1	0	0	0	1	0	1	0	1	1	0	0	0	0	28.1	22	13	44	33	33	21
		FULL2 23/33_1/2X	6.5	0	0	0	0	0	0	0	0	1	0	0	0	0	0	0	0	0	0	1	0	0	0	0	0	0	0	1	1	0	0	0	0	12.5	11	0	11	33	11	14
		FULL2 23/33_1/4X	3.25	0	0	0	0	0	0	0	0	0	0	0	0	0	0	0	0	0	0	0	0	0	0	0	0	0	0	1	1	0	0	0	0	6.25	0	0	0	33	0	14
		FULL2 23/33_1/8X	1.625	0	0	0	0	0	0	0	0	0	0	0	0	0	0	0	0	0	0	0	0	0	0	0	0	0	0	0	0	0	0	0	0	0	0	0	0	0	0	0
		FULL2 23/33_1/16X	0.8125	0	0	0	0	0	0	0	0	0	0	0	0	0	0	0	0	0	0	0	0	0	0	0	0	0	0	0	0	0	0	0	0	0	0	0	0	0	0	0
		FULL2 23/33_1/32X	0.40625	0	0	0	0	0	0	0	0	0	0	0	0	0	0	0	0	0	0	0	0	0	0	0	0	0	0	0	0	0	0	0	0	0	0	0	0	0	0	0
	Bait - Control	FULL2 19/37_1X	13	0	1	0	1	0	0	0	0	0	1	0	0	0	0	0	0	0	0	0	0	0	0	0	0	0	0	0	0	0	0	0	0	9.38	22	13	0	0	11	7.1
		FULL2 19/37_1/2X	6.5	0	0	0	1	0	0	0	0	0	0	0	0	0	0	0	0	0	0	0	0	0	0	0	0	0	0	0	0	0	0	0	0	3.13	11	0	0	0	5.6	0
		FULL2 19/37_1/4X	3.25	0	0	0	0	0	0	0	0	0	0	0	0	0	0	0	0	0	0	0	0	0	0	0	0	0	0	0	0	0	0	0	0	0	0	0	0	0	0	0
		FULL2 19/37_1/8X	1.625	0	0	0	0	0	0	0	0	0	0	0	0	0	0	0	0	0	0	0	0	0	0	0	0	0	0	0	0	0	0	0	0	0	0	0	0	0	0	0
		FULL2 19/37_1/16X	0.8125	0	0	0	0	0	0	0	0	0	0	0	0	0	0	0	0	0	0	0	0	0	0	0	0	0	0	0	0	0	0	0	0	0	0	0	0	0	0	0
		FULL2 19/37_1/32X	0.40625	0	0	0	0	0	0	0	0	0	0	0	0	0	0	0	0	0	0	0	0	0	0	0	0	0	0	0	0	0	0	0	0	0	0	0	0	0	0	0
		FULL2 23/37_1X	13	0	0	0	0	0	0	0	0	0	0	0	0	0	0	1	0	0	0	0	0	0	0	1	0	0	1	0	0	0	0	0	0	9.38	0	13	22	0	11	7.1
		FULL2 23/37_1/2X	6.5	0	0	0	0	0	0	0	0	0	0	0	0	0	0	1	0	0	0	0	0	0	0	1	0	0	0	0	0	0	0	0	0	6.25	0	13	11	0	5.6	7.1
		FULL2 23/37_1/4X	3.25	0	0	0	0	0	0	0	0	0	0	0	0	0	0	0	0	0	0	0	0	0	0	0	0	0	0	0	0	0	0	0	0	0	0	0	0	0	0	0
		FULL2 23/37_1/8X	1.625	0	0	0	0	0	0	0	0	0	0	0	0	0	0	0	0	0	0	0	0	0	0	0	0	0	0	0	0	0	0	0	0	0	0	0	0	0	0	0
		FULL2 23/37_1/16X	0.8125	0	0	0	0	0	0	0	0	0	0	0	0	0	0	0	0	0	0	0	0	0	0	0	0	0	0	0	0	0	0	0	0	0	0	0	0	0	0	0
		FULL2 23/37_1/32X	0.40625	0	0	0	0	0	0	0	0	0	0	0	0	0	0	0	0	0	0	0	0	0	0	0	0	0	0	0	0	0	0	0	0	0	0	0	0	0	0	0

Figure S8a-b

c

Genomic Loci	Interaction (F/R)	Primer Set and Dilution	DNA Input (ng)	Quality controlled Sample Data																																Detection rates (%)						
				CML (Bone Marrow)									Control (Bone Marrow)								CML (Peripheral Blood)									Control (Peripheral Blood)												
				AD60	AD61	AD166	AD171	AD173	AD202	AD206	AD212	AD241	Knee 8	Knee 9	Knee 10	Knee 11	Knee 12	Knee 13	Knee 14	Knee 15	AD035	AD096	AD256	AD264	AD277	AD290a	AD294	AD301a	AD315	AD272	AD276	AD289	AD316	AD334	AD346	Sample detection (%)	CML BM (%)	Control BM (%)	CML PB (%)	Control PB (%)	Total CML (%)	Total Control (%)
TP53	Bait - Hit 1	FULL2 3/13_1X	13	1	1	1	1	1	1	1	1	1	1	1	1	1	1	1	1	1	0	1	1	1	1	1	1	1	1	1	1	1	1	1	1	97	100	100	89	100	94	100
		FULL2 3/13_1/2X	6.5	0	1	1	1	1	1	1	1	1	1	1	1	0	1	1	1	0	0	1	0	1	1	1	1	1	1	1	1	0	1	1	1	81	89	75	78	83	83	79
		FULL2 3/13_1/4X	3.25	0	1	1	0	1	0	0	1	1	0	1	0	0	1	0	0	0	0	0	0	0	1	1	0	1	1	1	1	0	1	0	0	44	56	25	44	50	50	36
		FULL2 3/13_1/8X	1.625	0	0	1	0	1	0	0	0	1	0	0	0	0	1	0	0	0	0	0	0	0	0	0	0	1	0	1	1	0	1	0	0	25	33	13	11	50	22	29
		FULL2 3/13_1/16X	0.8125	0	0	1	0	0	0	0	0	0	0	0	0	0	0	0	0	0	0	0	0	0	0	0	0	1	0	0	0	0	0	0	0	6	11	0	11	0	11	0
		FULL2 3/13_1/32X	0.40625	0	0	0	0	0	0	0	0	0	0	0	0	0	0	0	0	0	0	0	0	0	0	0	0	0	0	0	0	0	0	0	0	0	0	0	0	0	0	0
	Bait - Hit 2	FULL2 3/9_1X	13	1	1	1	1	1	1	1	1	1	1	1	0	1	1	1	0	1	1	1	1	1	1	1	1	1	1	1	1	1	1	0	1	91	100	75	100	83	100	79
		FULL2 3/9_1/2X	6.5	1	0	1	1	1	1	0	0	1	0	1	0	1	1	1	0	1	1	1	1	1	1	1	1	1	0	1	0	1	1	0	1	72	67	63	89	67	78	64
		FULL2 3/9_1/4X	3.25	1	0	1	1	1	1	0	0	0	0	1	0	1	0	1	0	1	1	1	1	0	0	1	0	1	0	1	0	0	0	0	0	47	56	50	56	17	56	36
		FULL2 3/9_1/8X	1.625	0	0	1	1	0	1	0	0	0	0	0	0	1	0	1	0	0	0	1	1	0	0	1	0	0	0	0	0	0	0	0	0	25	33	25	33	0	33	14
		FULL2 3/9_1/16X	0.8125	0	0	1	1	0	0	0	0	0	0	0	0	1	0	1	0	0	0	0	1	0	0	1	0	0	0	0	0	0	0	0	0	19	22	25	22	0	22	14
		FULL2 3/9_1/32X	0.40625	0	0	0	0	0	0	0	0	0	0	0	0	0	0	0	0	0	0	0	0	0	0	0	0	0	0	0	0	0	0	0	0	0	0	0	0	0	0	0
	Bait - Control	FULL2 3/41_1X	13	0	0	0	0	0	0	0	0	0	0	0	1	1	0	0	0	1	0	0	1	1	0	0	0	1	0	0	0	0	0	0	0	19	0	38	33	0	17	21
		FULL2 3/41_1/2X	6.5	0	0	0	0	0	0	0	0	0	0	0	0	0	0	0	0	1	0	0	1	0	0	0	0	0	0	0	0	0	0	0	0	6	0	13	11	0	6	7
		FULL2 3/41_1/4X	3.25	0	0	0	0	0	0	0	0	0	0	0	0	0	0	0	0	0	0	0	0	0	0	0	0	0	0	0	0	0	0	0	0	0	0	0	0	0	0	0
		FULL2 3/41_1/8X	1.625	0	0	0	0	0	0	0	0	0	0	0	0	0	0	0	0	0	0	0	0	0	0	0	0	0	0	0	0	0	0	0	0	0	0	0	0	0	0	0
		FULL2 3/41_1/16X	0.8125	0	0	0	0	0	0	0	0	0	0	0	0	0	0	0	0	0	0	0	0	0	0	0	0	0	0	0	0	0	0	0	0	0	0	0	0	0	0	0
		FULL2 3/41_1/32X	0.40625	0	0	0	0	0	0	0	0	0	0	0	0	0	0	0	0	0	0	0	0	0	0	0	0	0	0	0	0	0	0	0	0	0	0	0	0	0	0	0

Figure S8c

a

Primer set	CML										Other patients							
	Peripheral Blood								Bone Marrow		Peripheral Blood						Bone Marrow	
	AD 35	AD 35a*	AD 96	AD 256	AD 294	AD 301a	AD 454	AD 548	AD 454	AD 548	AD 278	AD 283	AD 419	AD 446	AD 394	AD 412	AD 289	AD 339
c-MYC Bait-Hit 1	0/3	1/3	2/3	1/3	2/3	0/3	3/3	3/3	0/3	0/3	3/3	3/3	2/3	3/3	0/3	2/3	3/3	3/3
c-MYC Bait-Hit 2-1	1/3	3/3	3/3	3/3	2/3	3/3	3/3	2/3	2/3	1/3	3/3	3/3	3/3	2/3	3/3	2/3	1/3	3/3
c-MYC Bait-Hit 2-2	3/3	3/3	3/3	3/3	3/3	2/3	3/3	3/3	2/3	1/3	2/3	2/3	2/3	3/3	3/3	1/3	3/3	3/3
Nega-tive contro l 1	0/3	2/3	1/3	0/3	0/3	1/3	0/3	1/3	0/3	0/3	1/3	0/3	1/3	1/3	0/3	1/3	1/3	2/3

b

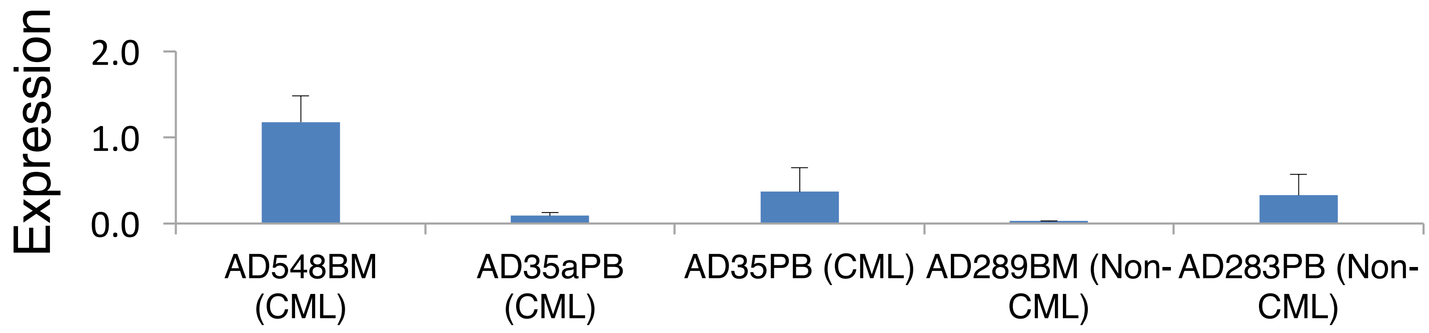

c

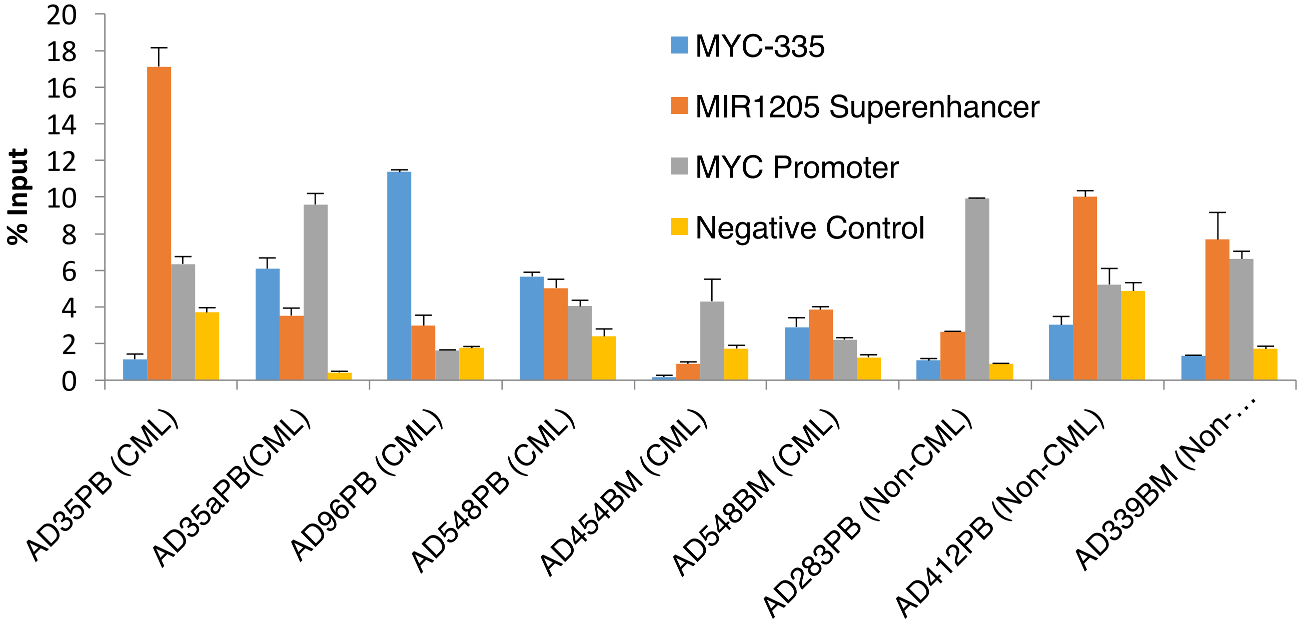

Figure S9

a

count of broad domains						
	DSE	PSE	DTE	PTE	Non-overlapping	Total
DBD	24	11	49	2	1	87
PBD	0	376	4	533	0	910
DTD	246	137	3473	187	2271	6314
PTD	4	564	39	9362	2673	12642

b

Count of super-enhancers						
	DBD	PBD	DTD	PTD	Non-overlapping	Total
DSE	22	0	151	4	5	164
PSE	11	376	109	428	1	752
DTE	49	4	3289	38	2842	6207
PTE	2	534	182	8955	713	10199

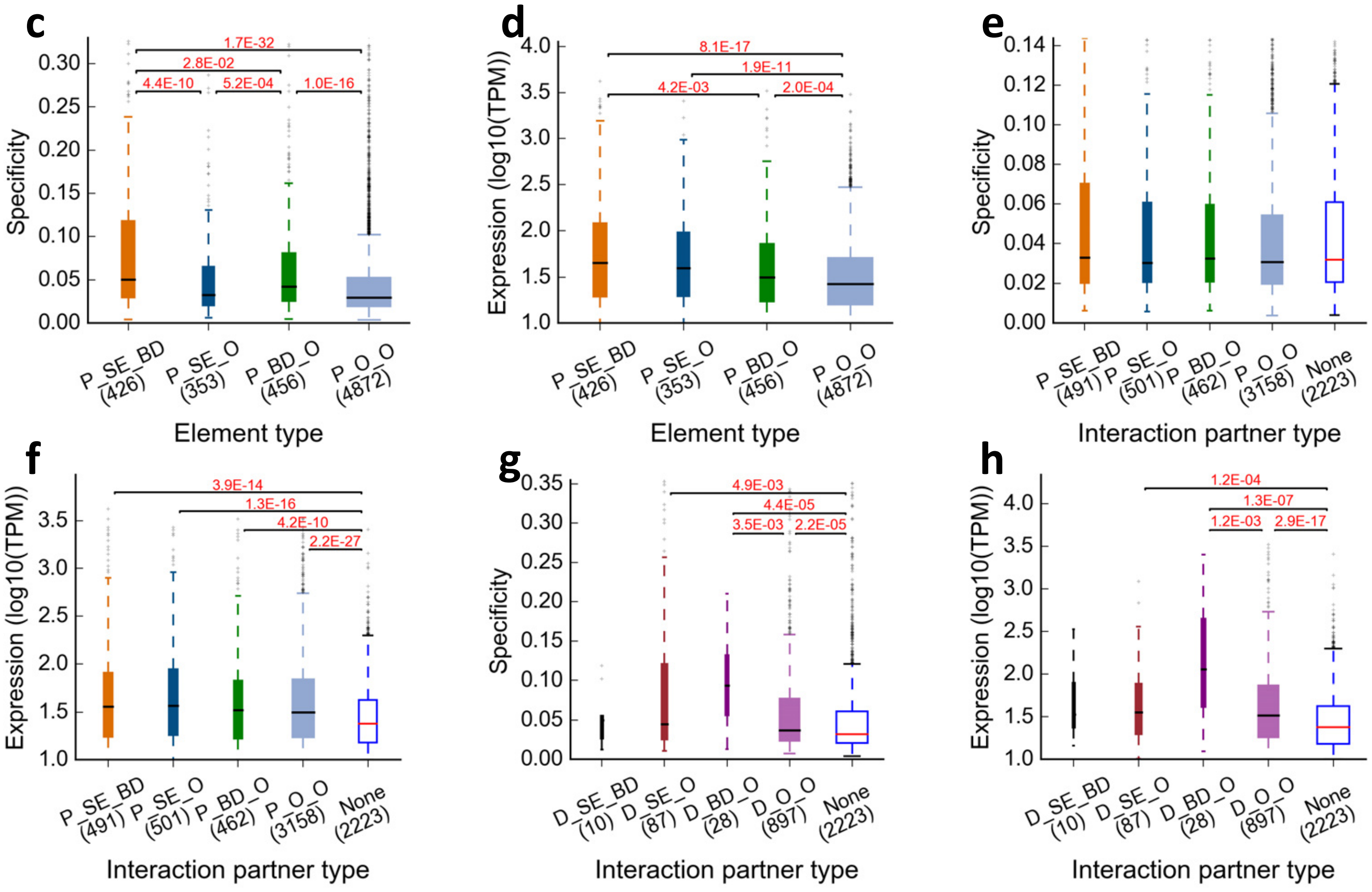

Figure S10

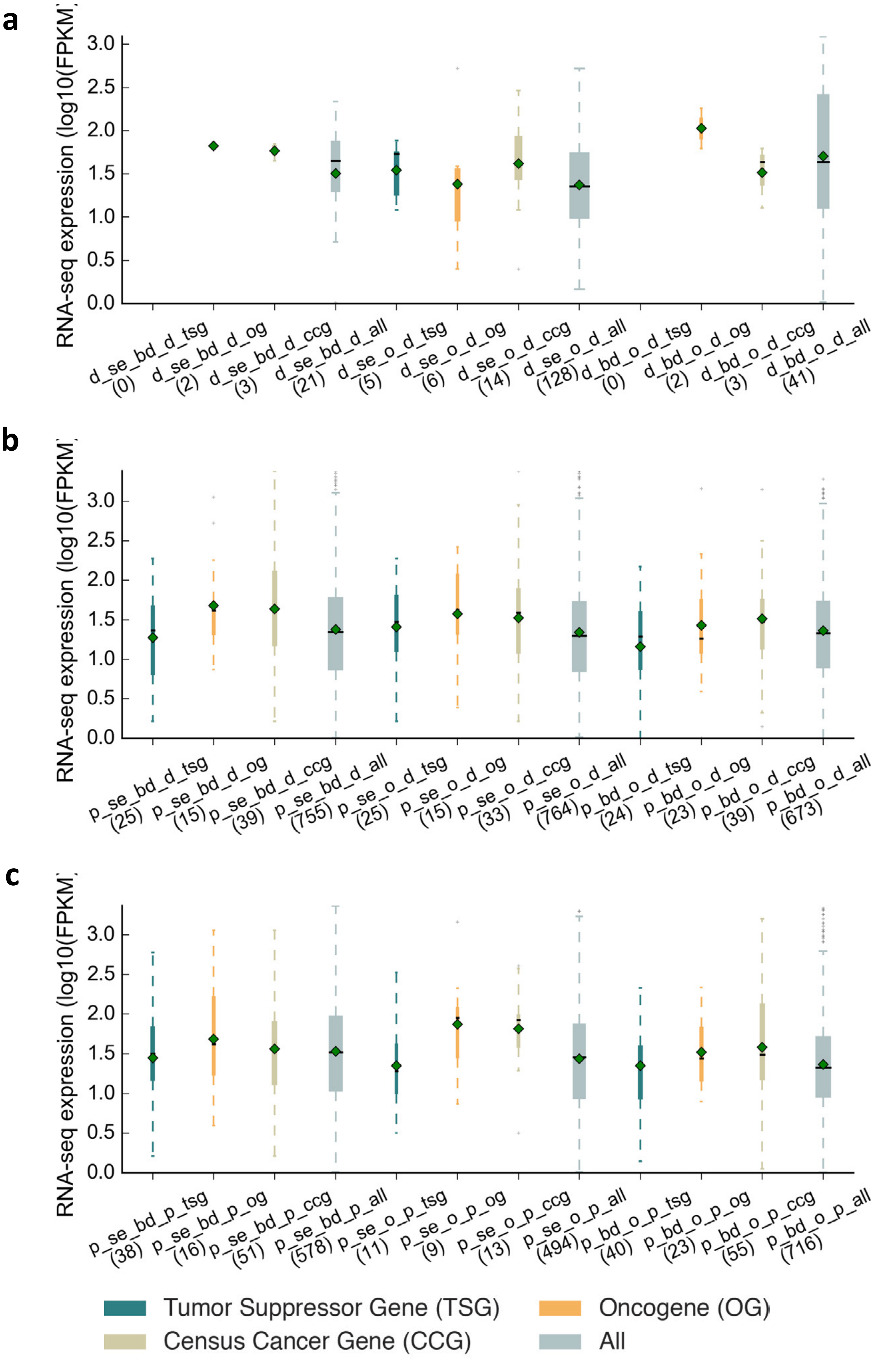

a
b
c
RNA-seq expression (log10(FPKM)
Tumor Suppressor Gene (TSG)
Oncogene (OG)
Census Cancer Gene (CCG)
All

Figure S11

a

count of broad domains						
	DSE	PSE	DTE	PTE	Non-overlapping	Total
DBD	4	1	11	1	9	26
PBD	1	62	6	1049	148	1264
DTD	115	85	1783	96	7339	9418
PTD	2	108	39	5945	9007	15101

b

Count of super-enhancers						
	DBD	PBD	DTD	PTD	Non-overlapping	Total
DSE	4	1	81	2	59	147
PSE	1	61	57	78	22	219
DTE	11	6	1725	39	4917	6698
PTE	1	1047	92	5746	1216	8102

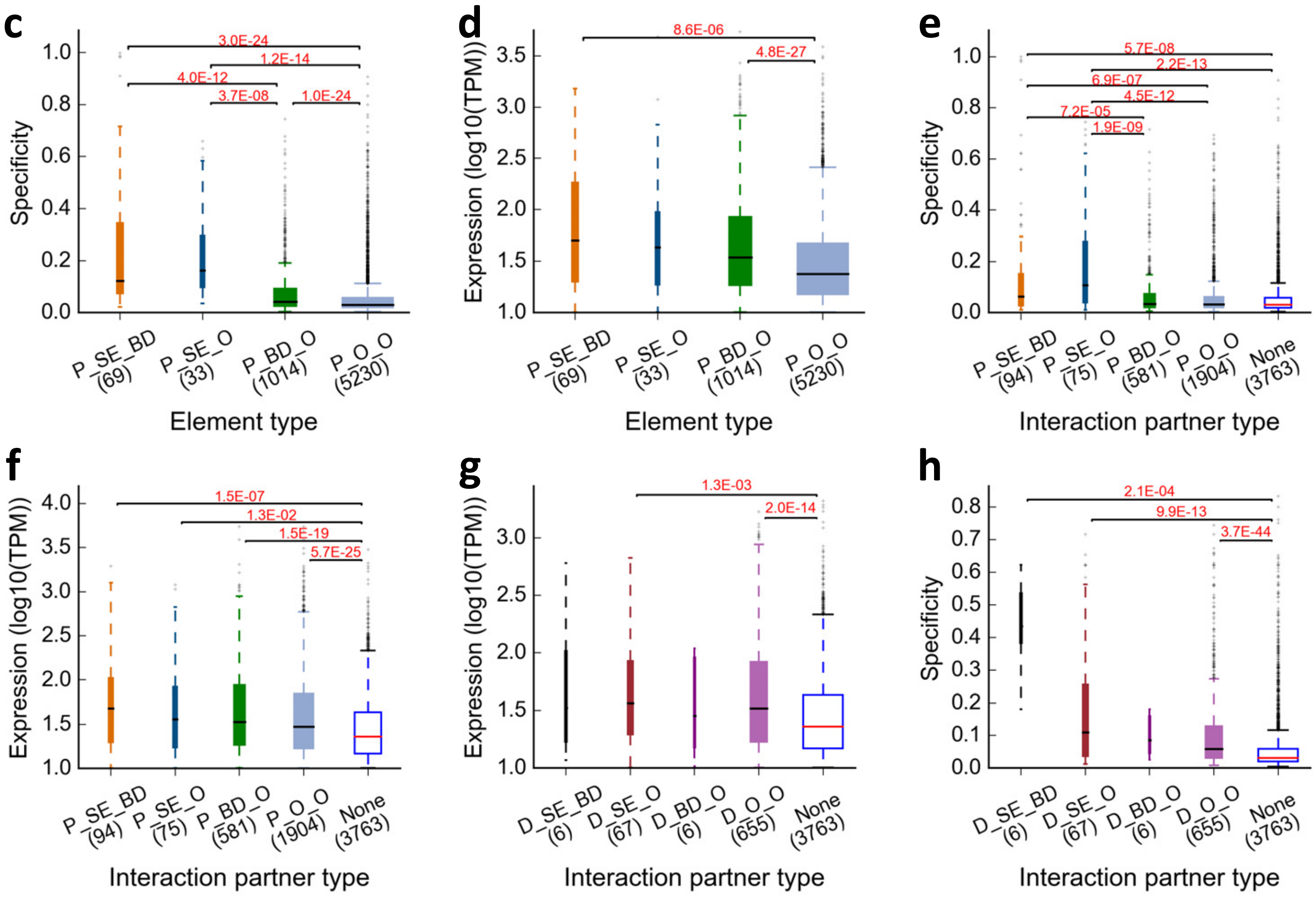

Figure S12

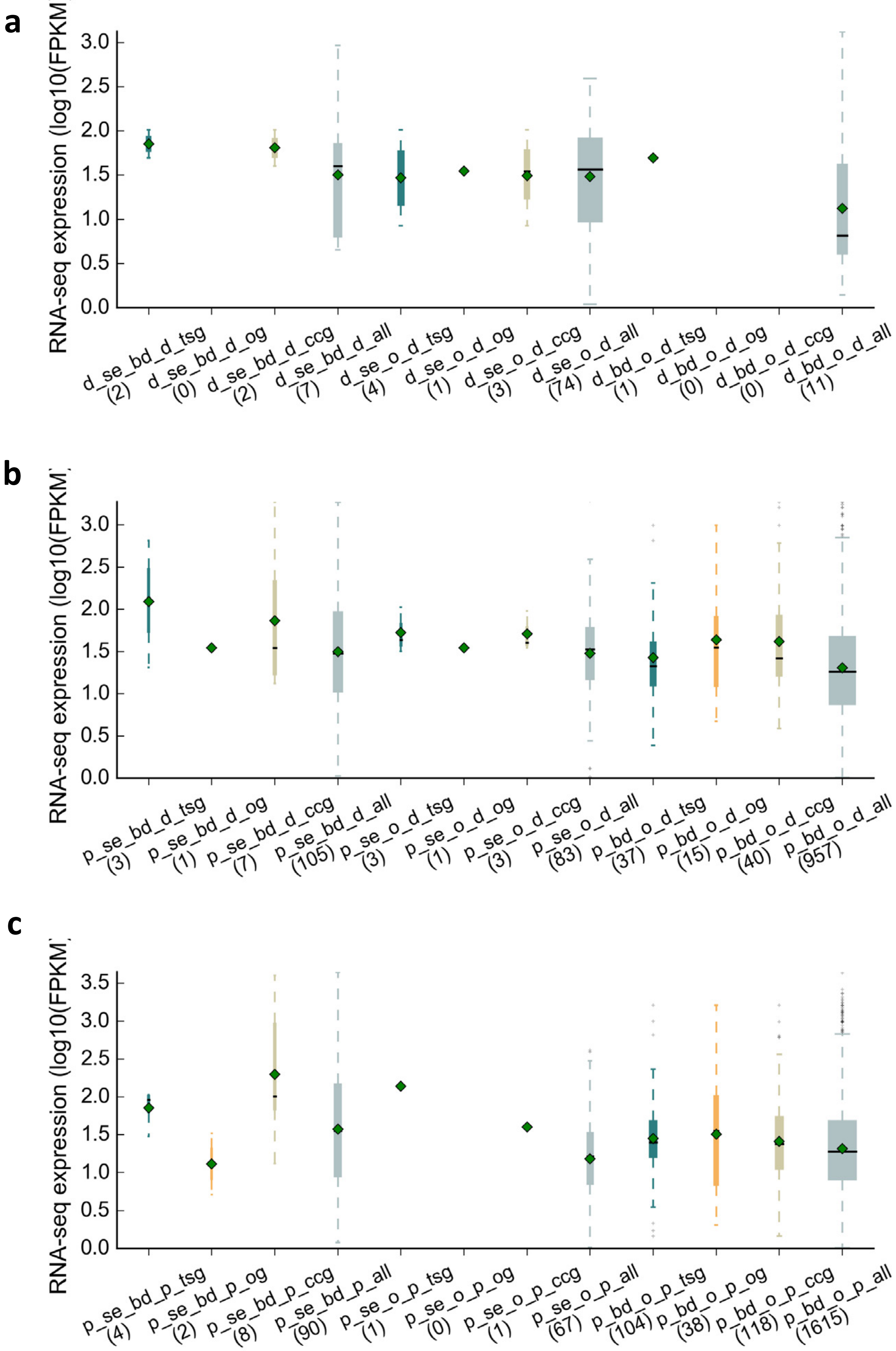
a
RNA-seq expression (log10(FPKM
d_se_bd_d_tsg (2)
d_se_bd_d_og (0)
d_se_bd_d_ccg (2)
d_se_bd_d_all (7)
d_se_o_d_tsg (4)
d_se_o_d_og (1)
d_se_o_d_ccg (3)
d_se_o_d_all (74)
d_bd_o_d_tsg (1)
d_bd_o_d_og (0)
d_bd_o_d_ccg (0)
d_bd_o_d_all (11)
b
RNA-seq expression (log10(FPKM
p_se_bd_d_tsg (3)
p_se_bd_d_og (1)
p_se_bd_d_ccg (7)
p_se_bd_d_all (105)
p_se_o_d_tsg (3)
p_se_o_d_og (1)
p_se_o_d_ccg (3)
p_se_o_d_all (83)
p_bd_o_d_tsg (37)
p_bd_o_d_og (15)
p_bd_o_d_ccg (40)
p_bd_o_d_all (957)
c
RNA-seq expression (log10(FPKM
p_se_bd_p_tsg (4)
p_se_bd_p_og (2)
p_se_bd_p_ccg (8)
p_se_bd_p_all (90)
p_se_o_p_tsg (1)
p_se_o_p_og (0)
p_se_o_p_ccg (1)
p_se_o_p_all (67)
p_bd_o_p_tsg (104)
p_bd_o_p_og (38)
p_bd_o_p_ccg (118)
p_bd_o_p_all (1615)

Figure S13

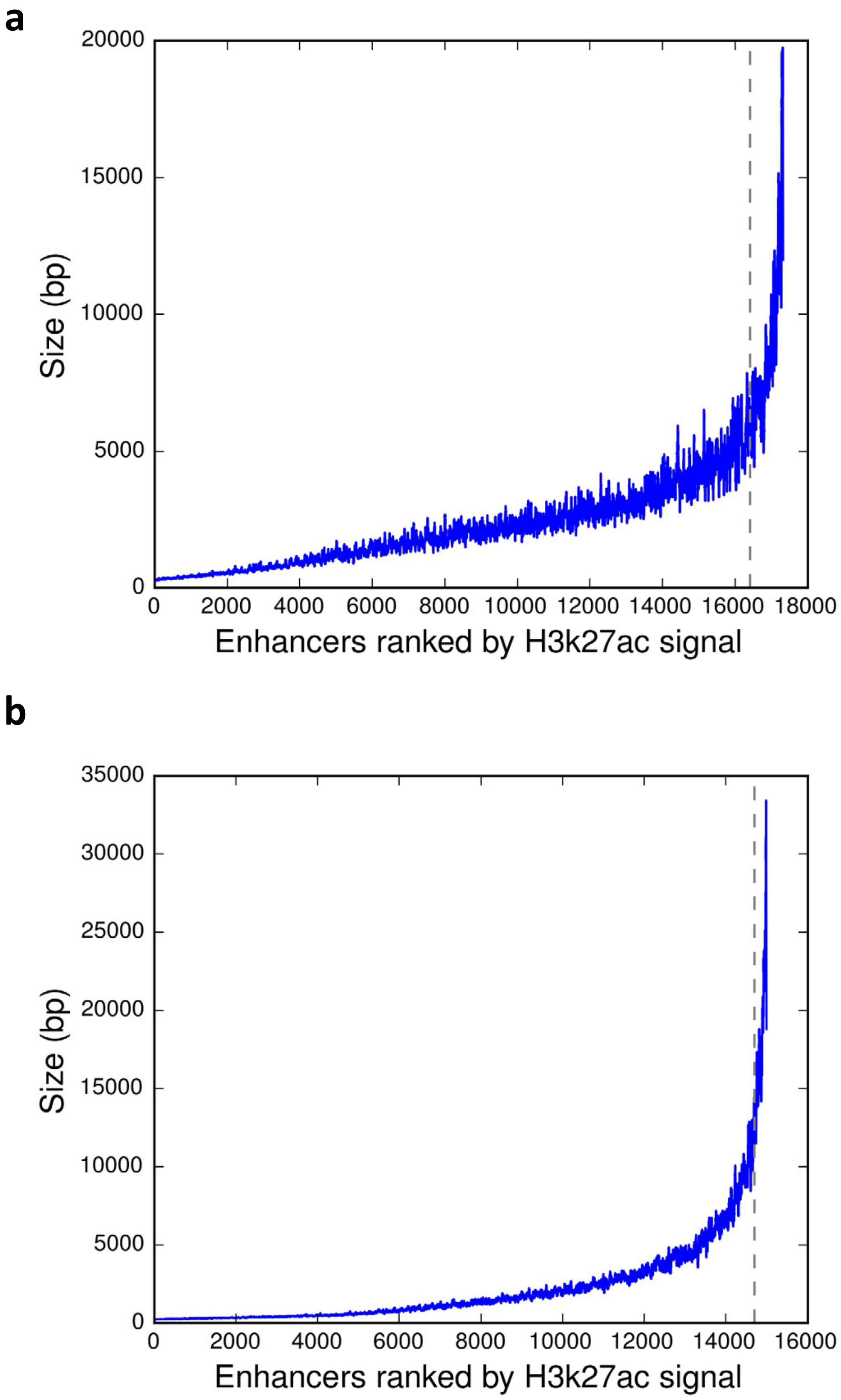
a
Size (bp)
Enhancers ranked by H3k27ac signal
0
5000
10000
15000
20000
0
2000
4000
6000
8000
10000
12000
14000
16000
18000
b
Size (bp)
Enhancers ranked by H3k27ac signal
0
5000
10000
15000
20000
25000
30000
35000
0
2000
4000
6000
8000
10000
12000
14000
16000

Figure S14
